# Digyalipopeptide A, an antiparasitic cyclic peptide from the Ghanaian *Bacillus* sp. strain DE2B

**DOI:** 10.3762/bjoc.18.185

**Published:** 2022-12-28

**Authors:** Adwoa P Nartey, Aboagye K Dofuor, Kofi B A Owusu, Anil S Camas, Hai Deng, Marcel Jaspars, Kwaku Kyeremeh

**Affiliations:** 1 Marine and Plant Research Laboratory of Ghana, Department of Chemistry, University of Ghana, P.O. Box LG 56 Legon-Accra, Ghana; 2 Department of Biochemistry, Cell and Molecular Biology, University of Ghana, P.O. Box LG 54 Legon-Accra, Ghana; 3 Department of Biological, Physical and Mathematical Sciences, University of Environment and Sustainable Development, PMB, Somanya, Ghana; 4 Department of Parasitology, Noguchi Memorial Institute for Medical Research, University of Ghana, P.O. Box LG 581, Legon-Accra, Ghana; 5 Department of Biomedical Engineering, Faculty of Engineering, University of Samsun, Ballica Campus 55420, Samsun, Turkey; 6 Marine Biodiscovery Centre, Department of Chemistry, University of Aberdeen, Aberdeen AB24 3UE, Scotland, UK

**Keywords:** Leishmania, lipopeptides, molecular networking, sequence tagging, trypanosomes

## Abstract

During the continued isolation of different bacteria from highly diverse, low human activity environments in Ghana and the subsequent characterization and biological activity studies of their secondary metabolites, we found both Gram-positive and Gram-negative *Bacillus* strains to be ubiquitous and widespread. One of such strains, the Ghanaian novel *Bacillu*s sp. strain DE2B was isolated from rhizosphere soils collected from the Digya National Park in Ghana. Chromatographic purifications of the fermented culture extract of the strain DE2B, led to the isolation of a cyclic lipopeptide, digyalipopeptide A (**1**). Using 1D and 2D NMR data, mass spectrometry sequence tagging, advanced Marfey’s analysis, and the GNPS molecular networking we solved the full structure of digyalipopeptide A (**1**). We found that compound **1** is a member of a somewhat homologous series of peptides produced as a mixture by the strain containing the same amino acid sequence in the cyclic peptide backbone but differing only by the length of aliphatic fatty acid side chains. When tested against *Trypanosoma brucei* subsp. *brucei* strain GUTat 3.1 and *Leishmania donovani* (Laveran and Mesnil) Ross (D10), digyalipopeptide A (**1**) gave IC_50_ values of 12.89 µM (suramin IC_50_ 0.96 µM) and 4.85 µM (amphotericin B IC_50_ 4.87 µM), respectively. Furthermore, digyalipopeptide A (**1**) produced IC_50_ values of 10.07 µM (ampicillin IC_50_ 0.18 µM) and 10.01 µM (ampicillin IC_50_ 1.53 µM) for *Staphylococcus aureus* and *Shigella sonnei*, respectively. The selectivity and toxicity profile of compound **1** was investigated using normal cell lines, macrophages RAW 264.7. When tested against normal macrophages, compound **1** gave an IC_50_ value of 71.32 μM. Selectivity indices (SI) were obtained by calculating the ratio of the IC_50_ in RAW 264.7 to the IC_50_ in the respective microbe and neglected parasite. In the presence of RAW 264.7 cell lines, compound **1** was particularly selective towards *Leishmania donovani* (Laveran and Mesnil) Ross (D10) with an SI value of 14.71. The bioactivity studies conducted confirm the role of these cyclic lipopeptides as defense chemicals in their natural environment and their ability to be biologically active across different species.

## Introduction

Microbes belonging to the genus *Bacillus* are mainly Gram-positive with only a few examples characterized as Gram-negative [1]. *Bacillus* demonstrates a variety of colony morphologies and is ubiquitous in nature, being omnipresent in aquatic, benthic, halophilic, soil, and pathogenic environments [2]. Together, this group constitutes one of the most genetically and environmentally diverse genera in the microbial world [3]. The major secondary metabolic pathways, defense mechanisms, and intracellular transport processes vary widely among *Bacillus* depending on their natural habitats [4–6]. Although the majority of *Bacillus* species are non-pathogenic, the few well-studied and genome-sequenced pathogenic strains among the group have instilled fear in the scientists that may wish to investigate their potential. This has rendered the genera much less studied when compared to their *Streptomyces* counterparts in terms of bioprospecting for novel drug scaffolds. However, a substantial number of *Bacillus* species have proven to be of clinical, health, industrial research and agrochemical importance [7–12].

Interestingly, the resistance of *Bacillus* spores to heat, radiation, and disinfection coupled with an inherent biosynthetic potential that enables them to grow even in the presence of antibiotics such as nystatin and nalidixic acid makes it easier to isolate them from a number of soil and sediment types using the spread plate method (SPM) in our laboratory. Therefore, in our continued research on the discovery of new bioactive compounds from Ghanaian microbes, we have started to study interesting strains belonging to *Bacillus* genus. Previously, we reported a Ghanaian *Paenibacillus* sp. strain DE2SH which produced the novel antiparasitics paenidigyamycins A–J and digyaindoleacid A [13–15].

Herein, we report a novel Ghanaian *Bacillus* sp. strain DE2B with GenBank Accession Number: MN700169 (Figures S1 and S2 in Supporting Information File 1), isolated from the Digya National Park in the Brong Ahafo Region of Ghana. We found this strain to produce a cyclic lipopeptide which we have named digyalipopeptide A (**1**).

## Results and Discussion

### Structure elucidation

Compound **1** (digyalipopeptide A) was isolated by repeated semi-preparative reversed-phase HPLC with diode array detection (0–400 nm) at retention time of 27.5 min (see Supporting Information File 1, Figures S3–S7). It was obtained as a white amorphous powder which was bright yellow when in solution. The molecular formula of **1** was determined to be C_51_H_89_N_7_O_13_ based on the high resolution electrospray ionization mass spectrometry (HRESIMSMS) at *m*/*z* 1008.6610 for [M + H]^+^ similar to the calculated value of *m*/*z* 1008.6591 with Δ = −1.9 ppm. The molecular formula indicated 11 degrees of unsaturation and the analyses of the ^13^C NMR spectrum acquired in DMSO-*d*_6_, together with the distortionless enhancement by polarization transfer (DEPT-135) and the ^1^H, ^13^C, multiplicity edited pulsed field gradient heteronuclear single quantum coherence (HSQC) spectra showed the presence of ten sp^2^-hybridized carbon resonances all of which were assigned to carbonyl carbons of seven amides, two carboxylic acids, and one ester. Also present were forty-one sp^3^-hybridized carbon signals which were assigned to fourteen methines, fifteen methylenes and twelve methyl groups

The ^1^H NMR spectrum exhibited signals at δ_H_ 4.19 (ov., 1H, H-2), 4.08 (dd, *J* = 8.8, 6.0 Hz, 1H, H-7), 4.53 (ov., 1H, H-12), 4.52 (ov., 1H, H-16), 4.18 (ov., 1H, H-22), 4.08 (dd, *J* = 8.8, 6.0 Hz, 1H, H-33), and 4.44 (ov., 1H, H-27) which were indicative of the presence of α-hydrogens belonging to amino acid residues typical for peptides. Using ^1^H,^1^H homonuclear correlation spectroscopy (COSY) and ^1^H,^13^C total correlation spectroscopy (HSQC-TOCSY), the individual spin systems within each amino acid residue were fully established.

Subsequently, seven amino acid residues were identified as glutamic acid, valine-1, aspartic acid, leucine-1, valine-2, leucine-2 and valine-3, see Table 1 and Tables S1 and S2 in Supporting Information File 1. The addition of carbonyl carbon HMBC data gave the complete amino acids which were further sequenced using LC–HRESIMS^n^ sequence tag data.

**Table 1:** ^1^H NMR and ^13^C NMR spectroscopic data for compound **1** in DMSO-*d*_6_.

Residue	#	δc, mult	δ_H,_ mult (*J* in Hz)		Residue	#	δc, mult	δ_H,_ mult (*J* in Hz)
		
glutamic acid	1	171.8, C			leucine-2	26	171.6, C	
	2	51.9, CH	4.19, ov.			27	50.4, CH	4.44, ov.
	3	27.3, CH_2_	1.91, ov. 1.77, ov.			28	41.6, CH_2_	1.42, ov.
	4	29.9, CH_2_	2.22, t (7.9)			29	24.1, CH	1.53, ov.
	5	174.0, C				30, 31	23.0, CH_3_	0.86, ov.
	1NH		7.82, d (7.2)			6NH		8.30, ov.
	OH		12.24, s					
		
valine-1	6	170.6, C			valine-3	32	170.6, C	
	7	58.2, CH	4.08, dd (8.8, 6.0)			33	58.2, CH	4.08, dd (8.8, 6.0)
	8	30.5, CH	1.96, ov.			34	30.5, CH	1.96, ov.
	9,10	17.9, CH_3_	0.75, d (6.8)			35, 36	17.9, CH_3_	0.75, d (6.8)
	2NH		7.77, ov.			7NH		8.26, ov.
		
aspartic acid	11	169.8, C			fatty acid	37	169.4, C	
	12	49.6, CH	4.53, ov.			38	40.4, CH_2_	2.41, ov. 2.38, ov.
	13	35.9, CH_2_	2.70, dd (7.2, 18) 2.59, dd (9.5, 18)			39	71.6, CH	5.00, m
	14	171.3, C				40	33.2, CH_2_	1.55, ov.
	3NH		8.17, m			41	28.5, CH_2_	1.21, ov.
	OH		12.24, s			42	28.8, CH_2_	1.21, ov.
						43	28.9, CH_2_	1.21, ov.
leucine-1	15	171.8, C				44	28.9, CH_2_	1.21, ov.
	16	50.6, CH	4.52, ov.			45	28.9, CH_2_	1.21, ov.
	17	41.6, CH_2_	1.42, m			46	28.9, CH_2_	1.21, ov.
	18	24.1, CH	1.53, ov.			47	26.7, CH_2_	1.22, ov.
	19,20	22.9, CH_3_	0.86, ov.			48	39.7, CH_2_	1.53, ov.
	4NH		7.68, d (8.6)			49	27.3, CH	1.49, ov.
						50, 51	23.0, CH_3_	0.86, ov.
valine-2	21	172.2, C						
	22	57.2, CH	4.18, ov.					
	23	29.5, CH	2.07, h (6.0)					
	24	19.0, CH_3_	0.84, ov.					
	25	18.9, CH_3_	0.84, ov.					
	5NH		8.26, ov.					

The ^1^H NMR data also showed the presence of diastereotopic methylene protons at δ_H_ 2.41, 2.38 (ov., 2H, H-38) which were characteristic for a –CH_2_– sandwiched between the carbonyl carbon of an amide and a hydroxylated methine proton δ_H_ 5.00 (m, 1H, H-39). Interestingly, this spin system expands to include protons at δ_H_ 1.55 (ov., 2H, H-40), 1.21 (ov., 2H, H-41), 1.21 (ov., 2H, H-42), 1.21 (ov., 2H, H-43), 1.21 (ov., 2H, H-44), 1.21 (ov., 2H, H-45), and 1.21 (ov., 2H, H-46) suggesting the presence of a long aliphatic chain. Careful study of the protons at δ_H_ 1.21 (ov., 2H, H-47), 1.53 (ov., 2H, H-48), 1.49 (ov., 1H, H-49), and 0.86 (ov., 6H, H-50, H-51) also suggested that the long aliphatic chain terminated as an isopropyl.

The COSY correlation H-38 (δ_H_ 2.41, 2.38)/H-39 (δ_H_ 5.00) which for this spin system is largely extended by the gHSQC-TOCSY correlations C-38 (δ_C_ 40.4)/H-39 (δ_H_ 5.00), C-39 (δ_C_ 71.6)/H-38 (δ_H_ 2.41, 2.38), H-40 (δ_H_ 1.55), H-41 (δ_H_ 1.21), H-42 (δ_H_ 1.21), H-43 (δ_H_ 1.21), H-44 (δ_H_ 1.21), H-45 (δ_H_ 1.21), H-46 (δ_H_ 1.21), H-48 (δ_H_ 1.21), C-40 (δ_C_ 33.2)/H-38 (δ_H_ 2.41, 2.38), H-39 (δ_H_ 5.00), H-41 (δ_H_ 1.21), H-42 (δ_H_ 1.21), H-43 (δ_H_ 1.21), H-44 (δ_H_ 1.21), H-45 (δ_H_ 1.21), H-46 (δ_H_ 1.21), C-42 (δ_C_ 28.8)/H-39 (δ_H_ 5.00), H-40 (δ_H_ 1.55), H-48 (δ_H_ 1.21), C-47 (δ_C_ 26.7)/H-41 (δ_H_ 1.21), H-42 (δ_H_ 1.21), H-43 (δ_H_ 1.21), H-44 (δ_H_ 1.21), H-45 (δ_H_ 1.21), H-46 (δ_H_ 1.21), and C-48 (δ_C_ 39.7)/H-50 (δ_H_ 0.86) fully confirms the aliphatic side chain terminating by an isopropyl moiety. Some of the HMBC correlations used to support this substructure are, H-38 (δ_H_ 2.41, 2.38)/C-39 (δ_C_ 71.6), H-39 (δ_H_ 5.00)/C-37 (δ_C_ 169.4), H-45 (δ_H_ 1.21)/C-47 (δ_C_ 26.7), H-46 (δ_H_ 1.21)/C-47 (δ_C_ 26.7), and H-47 (δ_H_ 1.22)/C-45 (δ_C_ 28.9).

**Figure 1:**
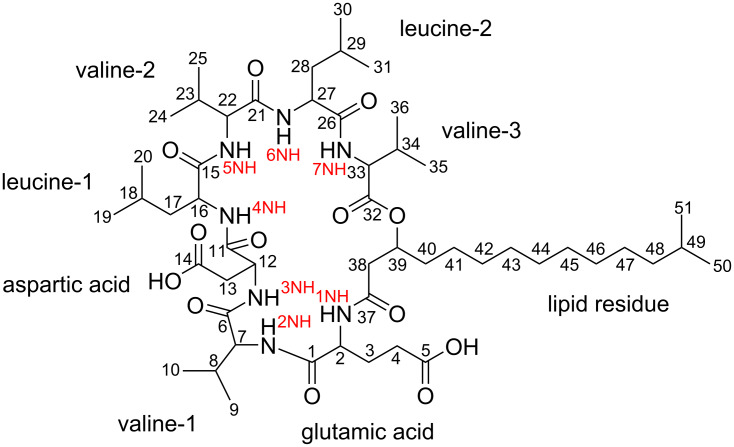
Primary structure of digyalipopeptide A (**1**).

**Figure 2:**
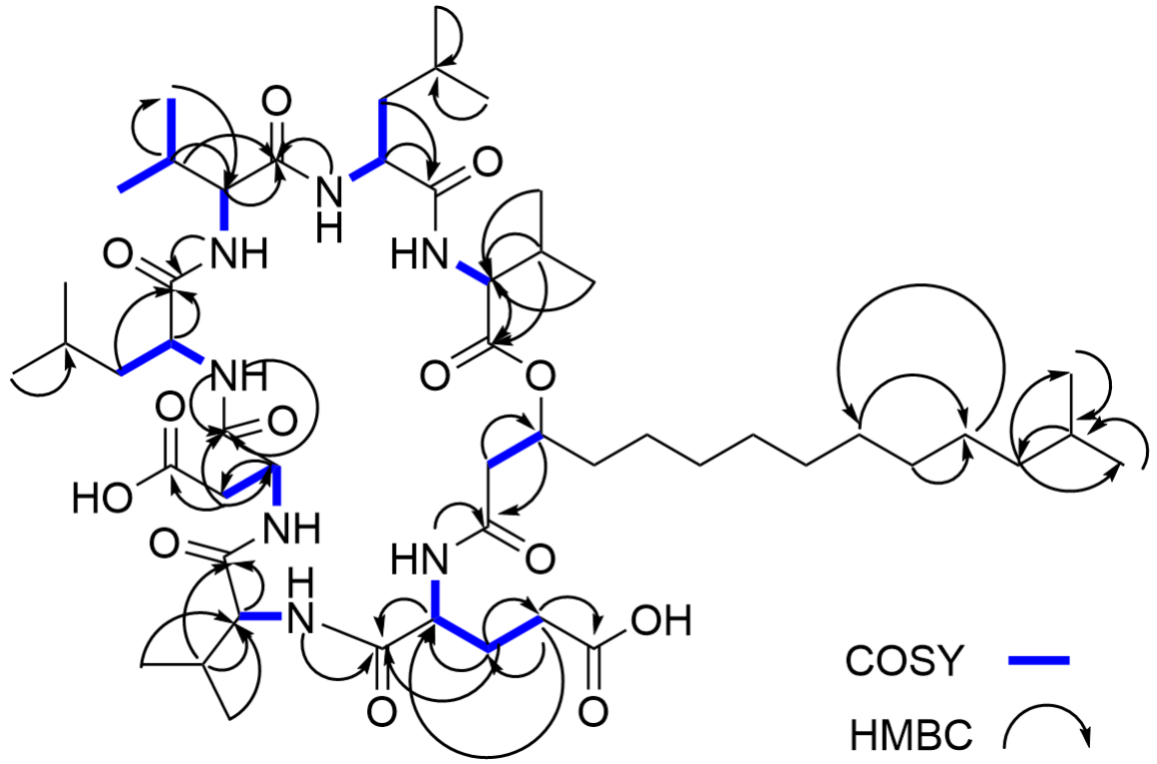
Key COSY and HMBC correlations for compound **1**.

**Figure 3:**
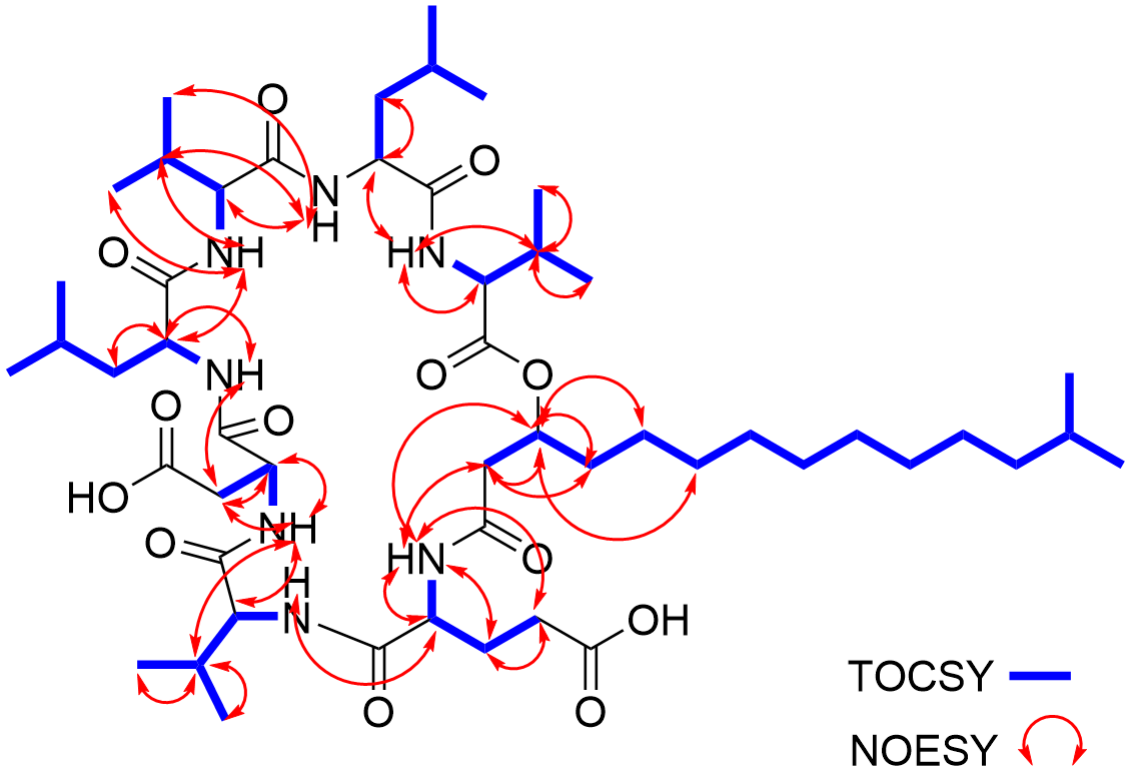
Key TOCSY and NOESY correlations for compound **1**.

The structure of compound **1** was further confirmed by the analysis of NOESY data, which along with the other 2D NMR data, are summarized in Figure 1, Figure 2 and Figure 3 and Table 1. The full details of all the NMR data with correlations can be found in Table S1 and Figures S22–S29 in Supporting Information File 1 but the summary for the data acquired in DMSO-*d*_6_ solvent is shown in Table 1. Subsequently, we dissolved compound **1** in deuterated chloroform, measured all 1D- and 2D NMR data and repeated the structure elucidation (see Supporting Information File 1, Table S2, and Figures S30–S37). The resultant structure confirmed the structural information initially obtained from the NMR data in DMSO-*d*_6_ solvent.

### High-resolution ESI mass spectrometry (HRESIMS) sequence tags

**Figure 4:**
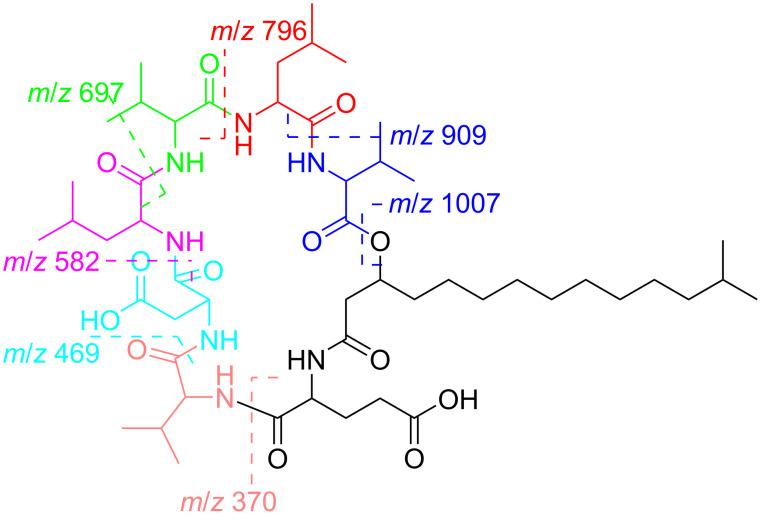
Liquid chromatography high resolution electrospray ionization mass spectrometry (LC–HRESIMS) sequence tags for compound **1**.

Furthermore, analysis of the LC–HRESIMS^n^ showed the presence of several *b* and *y* sequence tag ions indicating fragmentations from the middle of the cyclic peptide and most importantly the specific positions of each amino acid residue (Figure 4). Due to the presence of the free hydroxy groups of aspartic and glutamic acid and the elaborate system of aliphatic side chains, the sequence tag ions generated by systematic loss of amino acids were each subjected to several facile losses of H_2_O, –CH_3_, –C_2_H_2_O and CO resulting in the generation of several peaks in the LC–HRESIMS^n^ spectrum. The *b* and *y* ion fragmentation derived sequence tags corresponding to *m/z* 909 (− 99Val), 796 (− 99Val − 113Leu), 794 (− 99Val − 115Asp), 780 (− 99Val − 129Glu), 697 (− 99Val − 113Leu − 99Val), 582 (− 99Val − 113Leu − 99Val − 113Leu), 469 (− 99Val − 113Leu − 99Val − 113Leu − 115Asp) and 370 (− 99Val − 113Leu − 99Val − 113Leu − 115Asp − 99Val) provided the needed sequence information for the structure of compound **1**.

### Stereochemistry determination

Historically, the determination of the regiochemistry of enantiomeric amino acid residues within natural product peptides has proved challenging sometimes requiring the strenuous efforts of de novo total synthesis or degradation. This is because in natural product peptides both the ᴅ- and ʟ-forms of the different amino acids may be incorporated into the peptide structure. In order to determine the regiochemistry of the Val, Leu, Asp, and Glu amino acid residues in compound **1**, we deployed the advanced Marfey’s method [16–18] involving the complete hydrolysis of compound **1** followed by derivatization of the resultant amino acid residues with *N*-α-(2,4-dinitro-5-fluorophenyl)-ʟ-alanine amide (ʟ-FDAA).

**Figure 5:**
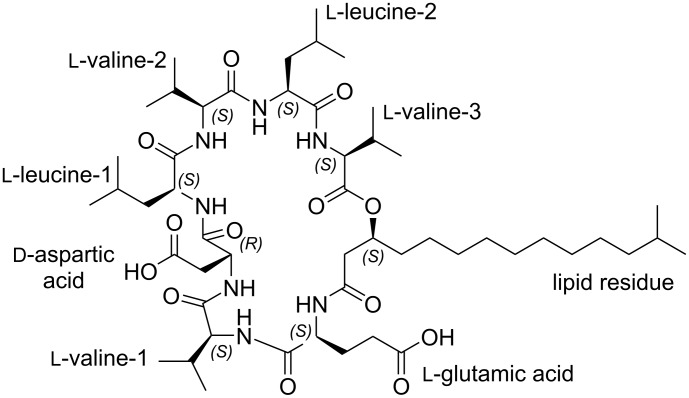
The absolute stereochemistry of compound **1**.

Similar derivatization reactions between ʟ-FDAA and authentic amino acid standards, followed by their injection on analytical reversed-phase HPLC-DAD in comparison to the naturally derived amino acid residue derivatives of **1** provided information on the absolute stereochemistry of the component amino acids in compound **1** (see Figure 5 and Figures S9–S13 and Table S3 in Supporting Information File 1).

However, it was difficult to determine the absolute stereochemistry around the esterified methine carbon C-39 (δ_C_ 71.6) of the fatty acid chain. In the absence of an authentic laboratory standard for the fatty acid chain coupled with the inability to obtain well-defined multiplicity patterns in the ^1^H NMR spectrum amidst huge overlaps of signals for δ_H_ 2.41, 2.38 (ov., 2H, H-38), δ_H_ 5.00 (m, 1H, H-39), δ_H_ 1.55 (ov., 2H, H-40) made it difficult to also obtain coupling constants (^3^*J*_HH_) for information on the relative spatial orientation of residues in this part of the structure.

**Figure 6:**
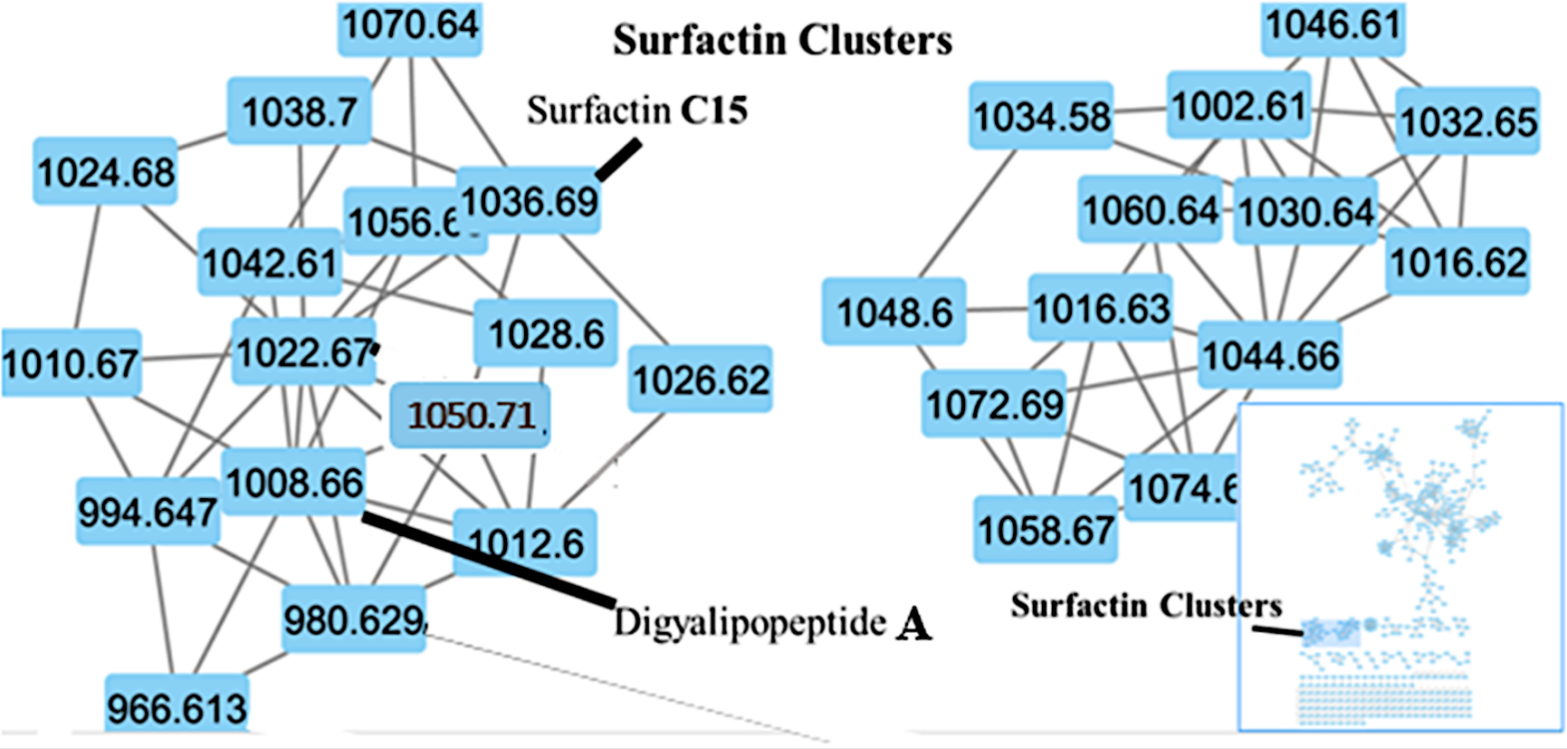
Global natural products social molecular networking (GNPS).

In order to confirm the presence of the cyclic ester and the possible absolute stereochemistry around C-39 (δ_C_ 71.6) of the fatty acid chain we first of all consulted the global natural products social molecular networking (GNPS) [19–21]. The GNPS network data (Figure 6) provided the opportunity to examine similar lipopeptide structures previously characterized in the literature that possessed both the fatty acid chain terminating as an isopropyl and adjacent to Glu residue connected by an amide bond. This detailed examination revealed that in the structure of these cyclic lipopeptides, the configuration of the methine carbon C-39 (δ_C_ 71.6) of the fatty acid chain is always opposite to that of the adjacent Glu residue connected by an amide bond with typical examples being the pumilacidin B and surfactin C [22]. Therefore, since the stereochemistry of the Glu residue was *S* we tentatively proposed that the absolute stereochemistry of the methine carbon C-39 (δ_C_ 71.6) of the fatty acid chain is most likely to be *R*. In order to verify this hypothesis, we used the many accumulated NOESY correlations to make relative deductions that established the stereochemistry of the C-39 (δ_C_ 71.6). Firstly, H-2 has an *S* configuration pointing into the plane of the screen and oriented away from us. In this configuration, H-2 shows NOESY correlations H-2/1NH and H-2/2NH suggesting that protons 1NH and 2-NH also orient similarly to H-2. Therefore, the NOESY correlation H-39/1NH shows that the H-39 proton must point away from us resulting in an *S* configuration at C-39 as opposed to and *R* configuration. Subsequently, we propose the 3D structure of compound **1** as shown in Figure 5.

Structurally, compound **1** belongs to the surfactin group of lipopeptides that exhibit huge chemical diversity through the alternate insertion of different amino acids, homologous extension and linear, isopropyl or isobutyl termination of fatty acid chains. Therefore, the biosynthesis of compound **1** is speculated to occur via a similar pathway like the surfactins, which is known to be modulated by four enzyme subunits SrfAA, SrfAB, SrfAC, and SrfAD on the surfactin synthetase complex [23–24]. We hope to confirm this similarity in the near future as we proceed to further study the whole genome sequence of strain DE2B.

Subsequently, using the fully elucidated structure of compound **1**, we noticed that this compound was part of a homologous series that included the reversed phase HPLC co-eluting compounds shown in Figure S5 (Supporting Information File 1).

### Biological activity

Generally, cyclic lipopeptides have an amphipathic physicochemical property that directly promotes their ability to be compatible with both hydrophobic and hydrophilic biological environments. Repeated studies on the bioactivity of lipopeptides have shown that the primary mechanism of action of these peptides involves interactions with the double layer of lipids and proteins that constitute the cell membrane [24–26]. This mechanism of action is important, since it promotes the ability of these ubiquitous cyclic lipopeptides to be effective and biologically active across different organism types. Hence, cyclic lipopeptides have been previously reported to possess antimicrobial, antifungal, antiviral, anticancer, and cytotoxic biological activities, but their ability to produce antiparasitic activity against neglected tropical parasites such as trypanosomes and leishmanias is largely underinvestigated.

Therefore, we studied the biological activity profile of compound **1** against *Trypanosoma brucei* subsp. *brucei* strain GUTat 3.1 and *Leishmania donovani* (Laveran and Mesnil) Ross (D10). Compound **1** showed promising antiparasitic activity against *Leishmania donovani* with IC_50_ of 4.85 µM compared to the laboratory standard amphotericin B with IC_50_ value of 4.87 µM (Table 2 and Figure S13 in Supporting Information File 1). When tested against *Trypanosoma brucei* subsp. *brucei* strain GUTat 3.1, compound **1** gave an IC_50_ value of 12.89 µM compared to the laboratory standard, suramin with IC_50_ of 0.96 µM (Table 2 and Figure S14 in Supporting Information File 1).

**Table 2:** Antiparasitic activity data for compound **1**.

	Parasite	IC_50_ (µM) ± SEM	Positive control	IC_50_ (µM)

compound **1**	*L. donovani*	4.85 **±** 0.19	amphotericin B	4.87 **±** 0.21
	*T. brucei brucei*	12.89 **±** 0.22	suramin	0.96 **±** 0.15

Furthermore, we investigated the biological activity profile of compound **1** against a number of Gram-negative and Gram-positive standard laboratory bacteria including *Escherichia coli*, *Staphylococcus aureus*, *Bacillus cereus*, *Shigella flexneri*, *Shigella sonnei*, *Salmonella paratyphi B*, *Salmonella enteritidis*, *Salmonella typhimurium*, and *Shigella dysenteriae* (Table 3). Compound **1** was found to possess promising antimicrobial properties in comparison to the laboratory standards ampicillin and amphotericin B. Compound **1** produced interesting biological activity against the Gram-negative bacteria *Shigella sonnei*, *Shigella flexneri* and the multidrug resistant Gram-positive *Staphylococcus aureu*s with IC_50_ of 10.01, 18.71, and 10.01 µM, respectively. The laboratory standard itself produced IC_50_ of 1.53 µM for *Shigella sonnei*, *Shigella flexneri* 1.76 µM, and *Staphylococcus aureus* 0.18 µM, respectively (Table 3).

**Table 3:** Antibacterial activity data for compound **1**.

	Microbe	IC_50_ (µM) ± SEM	Positive control	IC_50_ (µM) ± SEM

compound **1**	*E. coli*	>100 ± 0.31	ampicillin	10.44 ±0.18
	*S. typhimurium*	34.27 ± 0.25	ampicillin	1.27 ± 0.15
	*B. cereus*	38.35 ± 0.21	ampicillin	1.70 ± 0.19
	*S. flexneri*	18.71 ± 0.18	ampicillin	1.76 ± 0.21
	*S. paratyphi B*	27.90 ± 0.19	amphotericin B	1.53 ± 0.20
	*S. enteritidis*	>100 ± 0.34	ampicillin	0.76 ± 0.30
	*S. aureus*	10.07 ± 0.29	ampicillin	0.18 ± 0.16
	*S. sonnei*	10.01 ± 0.15	ampicillin	1.53 ± 0.19
	*S. dysenteriae*	19.72 ± 0.20	ampicillin	1.07 ± 0.22

The selectivity and toxicity profile of compound **1** was also investigated using normal cell lines, macrophages RAW 264.7. When tested against normal macrophages, compound **1** produced an IC_50_ value of 71.32 μM. Selectivity indices (SI) were obtained by calculating the ratio of the IC_50_ in RAW 264.7 to the IC_50_ in the respective microbe or neglected parasite. In comparison to RAW 264.7 cell line, compound **1** was particularly selective towards *Leishmania donovani* (Laveran and Mesnil) Ross (D10) with an SI value of 14.71. Other promising SI values of 2.08, 1.86, 3.81, and 2.26 were also recorded for *Salmonella typhimurium*, *Bacillus cereus*, *Shigella flexneri*, and *Salmonella paratyphi* B, respectively, as shown in Table 4 and Figure S15 in Supporting Information File 1.

**Table 4:** Cytotoxicity and selectivity profiles of compound **1** using normal macrophages.

Microbe	Activity in microbe or parasite [IC_50_ (µM)]) ± SEM	Selectivity Index (SI)

*E. coli*	>100 ± 0.29	<0.71
*S. typhimurium*	34.27 ± 0.21	2.08
*B. cereus*	38.35 ± 0.18	1.86
*S. flexneri*	18.71 ± 0.24	3.81
*S. paratyphi B*	27.90 ± 0.28	2.26
*S. enteritidis*	>100 ± 0.20	<0.71

Parasite		

*T. brucei brucei*	12.89 ± 0.19	5.53
*L. donovani*	4.85 ± 0.22	14.71

## Conclusion

In conclusion, a new cyclic lipopeptide has been isolated from the novel Ghanaian *Bacillus* sp. Strain DE2B. The structure of this peptide was established through detailed analysis of 1D and 2D NMR spectroscopic data acquired in DMSO-*d*_6_, HRESIMSMS sequence tagging data with GNPS molecular networking and the advanced Marfey’s test. We proposed that the biosynthesis of compound **1** is similar to that of other surfactin-like lipopeptides previously reported in the literature. We found compound **1** to possess remarkable antiparasitic activity against *Trypanosoma brucei* subsp. *brucei* strain GUTat 3.1 and *Leishmania donovani* (Laveran and Mesnil) Ross (D10).

Furthermore, the antimicrobial bioactivity of compound **1** was also evaluated against *Escherichia coli*, *Staphylococcus aureus*, *Bacillus cereus*, *Shigella flexneri*, *Shigella sonnei*, *Salmonella paratyphi B*, *Salmonella enteritidis*, *Salmonella typhimurium*, and *Shigella dysenteriae*. Compound **1** produced the best antimicrobial activity results against *Shigella sonnei*, *Shigella flexneri*, and the multidrug resistant Gram-positive *Staphylococcus aureu*s.

Subsequently, we reiterate the fact that it is important not to overlook *Bacillus* and their natural products especially in this current era of spreading antibiotic resistance and demands for ecologically friendly pest control strategies.

## Supporting Information

File 1 Experimental protocols, phylogenetic data, compound characterization data (1D, 2D NMR), stereochemistry determination, tables, and figures.
