# Supplementary material for: Digyalipopeptide A, an antiparasitic cyclic peptide from the Ghanaian Bacillus sp. strain DE2B
Source: Beilstein J Org Chem. 2022 Dec 28;18:1763–71. doi: 10.3762/bjoc.18.185 (PMC9811306; doi:10.3762/bjoc.18.185)
Supplement: File 1 — Experimental protocols, phylogenetic data, compound characterization data (1D, 2D NMR), stereochemistry determination, tables, and figures. [file Beilstein_J_Org_Chem-18-1763-s001.pdf]

BEILSTEIN JOURNAL OF ORGANIC CHEMISTRY

Supporting Information

for

Digyalipopeptide A, an antiparasitic cyclic peptide from the Ghanaian *Bacillus* sp. strain DE2B

Adwoa P. Nartey, Aboagye K. Dofuor, Kofi B. A. Owusu, Anil S. Camas, Hai Deng, Marcel Jaspars and Kwaku Kyeremeh

Beilstein J. Org. Chem. **2022,** *18,* 1763–1771. doi:10.3762/bjoc.18.185

Experimental protocols, phylogenetic data, compound characterization data (1D, 2D NMR), stereochemistry determination, tables, and figures

License and Terms: This is a supporting information file under the terms of the Creative Commons Attribution License (https://creativecommons.org/licenses/by/4.0). Please note that the reuse, redistribution and reproduction in particular requires that the author(s) and source are credited and that individual graphics may be subject to special legal provisions.
The license is subject to the *Beilstein Journal of Organic Chemistry* terms and conditions: (https://www.beilstein-journals.org/bjoc/terms)

Table of contents

Supplementary methods **S3**

General experimental procedures S3

Sediment sample collection site S3

Culture and isolation of strain DE2B from soil sediment S4

Identification of strain DE2B S5

Culture and fermentation of strain DE2B S5

Extraction and purification procedures S6

Physical data for compound 1 .... S6

Determination of absolute configuration for amino acid residues in the structure of compound 1 .... S7

Bioassay reagents S7

Preparation of compound for bioassay S8

Cell culture S8

In vitro viability test for trypanosomes and macrophages S9

In vitro viability test for Leishmania parasites S9

In vitro viability test for laboratory microbes S9

Supplementary tables **S10**

Table S1. 1D and 2D NMR spectroscopic data for compound 1 acquired in DMSO-d_6. .... S10

Table S2. 1D and 2D NMR spectroscopic data for compound 1 acquired in $CDCl_3$. S13

Table S3. Reverse Phase HPLC retention times (RT) for the standard amino acids (SAA) and the amino acids present in compound 1 (AAC1). S14

Supplementary figures **S15**

Figure S1: Pure *Bacillus* sp. strain DE2B growing on ISP2 agar plates at pH 5.5 and 28 °C incubation temperature. S15

Figure S2: Phylogenetic tree of Bacillus sp. DE2B. S16

Figure S3: Modified Kupchan solvent partitioning of the crude extract of Bacillus sp. DE2B gives FH, FD, FM, and WB fractions. S17

Figure S4: Sephadex LH-20 chromatography of FD fraction followed by reversed phase semi-preparative HPLC gives pure compound 1. .... S17

Figure S5: First HPLC chromatogram for FD-SFB. S18

Figure S6: Repeated HPLC chromatogram for compound 1 collected from the first HPLC in Figure S6. .... S19

Figure S7: Infrared spectrum of compound 1. .... S20

Figure S8: Reversed phase HPLC chromatogram for Marfey derivatised amino acid residues from compound 1. .... S20

Figure S9: Reversed phase HPLC chromatogram and UV profile of Marfey derivative for L-Glu residue. S21

Figure S10: Reversed phase HPLC chromatogram and UV profile of Marfey derivative for L-Val residue S21

Figure S11: Reversed phase HPLC chromatogram and UV profile of Marfey derivative for L-Leu residue. S22

Figure S12: Reversed phase HPLC chromatogram and UV profile of Marfey derivative for D-Asp residue. S22

Figure S13: The IC50 plot for the observed antileishmania activity of compound 1 using Leishmania donovani (Laveran and Mesnil) Ross (D10). S23

Figure S14: The IC50 plot for the observed antitrypanosomal activity of compound 1 using Trypanosoma brucei subsp. brucei strain GUTat 3.1. .... S23

Figure S15: IC_{50} plot showing cytotoxicity test of compound 1 using Raw 264.7 cell lines (macrophages). S24

Figure S16: HR-ESI-LC-MSMS data showing fragmentation pattern for compound 1 .... S25

Figure S17: HR-ESI-LC-MSMS data with sequence tag information for compound 1 .... S25

Figure S18: 500 MHz ^{1}H NMR spectrum of compound 1 in DMSO-d_6. .... S26

Figure S19: 500 MHz ^{13}C NMR spectrum of compound 1 in DMSO-d_6. .... S26

Figure S20: 500 MHz HSQC NMR spectrum of compound 1 in DMSO-d_6. .... S27

Figure S21: 500 MHz COSY NMR spectrum of compound 1 in DMSO-d_6. .... S27

Figure S22: 500 MHz HMBC NMR spectrum of compound 1 in DMSO-d_6. .... S28

Figure S23: 500 MHz gHSQC-TOCSY NMR spectrum of compound 1 in DMSO-d_6. .... S28

Figure S24: 500 MHz DEPT-135 NMR spectrum of compound 1 in DMSO-d_6. .... S29

Figure S25. 500 MHz ROESY NMR spectrum of compound 1 in DMSO-d_6. .... S29

Figure S26: 500 MHz ^{1}H NMR spectrum of compound 1 in $CDCl_3$. S30

Figure S27: 500 MHz ^{13}C NMR spectrum of compound 1 in $CDCl_3$. S30

Figure S28: 500 MHz HSQC spectrum of compound 1 in $CDCl_3$. S31

Figure S29: 500 MHz COSY spectrum of compound 1 in $CDCl_3$. S31

Figure S30: 500 MHz HMBC spectrum of compound 1 in $CDCl_3$. S32

Figure S31: 500 MHz gHSQC-TOCSY spectrum of compound 1 in $CDCl_3$. S32

Figure S32: 500 MHz ROESY spectrum of compound 1 in $CDCl_3$. S33

Figure S33: 500 MHz DEPT-135 spectrum of compound 1 in $CDCl_3$. S33

References S34

Supplementary methods

General experimental procedures

1D and 2D NMR analysis was performed on a Bruker Ascend 500 MHz (BRUKER, Sylvenstein, Germany) spectrometer. This instrument was optimized for ^{1}H observation with pulsing/decoupling of ^{13}C and ^{15}N, with 2H lock channels equipped with shielded *z*-gradients and cooled preamplifiers for ^{1}H and ^{13}C. Solvent signals, δ_H 2.50 and δ_C 39.52 ppm in DMSO-d_6 and δ_H 7.26 and δ_C 77.16 ppm in $CDCl_3$ were used as reference for ^{1}H and ^{13}C chemical shifts. High resolution mass spectrometry data were obtained using a Thermo Instrument MS system (LTQ XL/LTQ Orbitrap Discovery, UK) coupled to a Thermo Instruments HPLC system (Accela PDA detector, Accela PDA autosampler and Accela pump). Conditions used in data acquisition were: capillary voltage of 45 V, a flow rate of 40–50 arbitrary units of sheath gas, a mass range of 100–2000 amu (maximum resolution of 30,000), a capillary temperature of 320 °C, 4.5 kV of spray voltage and an auxiliary gas flow rate between 10–20 arbitrary units. The IR spectrum was measured using a Nexus 870 FT-IR, UK. Optical rotation value was measured by a Autopol IV automatic polarimeter (Rudolph Research Analytical, USA). Separations on HPLC were done using a reversed-phase Phenomenex (C-18, 5 µm, 100 Å, 250 × 6.10 mm, L × i.d.) column which is coupled to a Waters 1525 series binary pump (France), an in-line degasser and a column heater. Detection and monitoring was carried out on a 2998 Waters photodiode array (PDA) detector (France). All solvents used were of HPLC grade and purchased from VWR, UK.

Sediment sample collection site

The Ghanaian *Bacillus sp.* strain DE2B was isolated from soil sediments sampled within the Digya National Park (coordinates: 7°31'44.85" N and 0°036'48.15" W), a forest reserve which is home to a wide variety of organisms and located along the western coasts of the Lake Volta in the Bono-East region of Ghana. This sampling site is well noted for the predominance of many undisturbed natural habitats including wetlands which represent one of the most bio-diverse environments in the country.

Culture and isolation of strain DE2B from soil sediment

In a manner similar to the procedure from [1] the soil sediment coded DE2B was prepared for the isolation of bacteria strains by placing 5 g of the soil sample into a new sterile 50 mL falcon tube. About 10 mL of sterilized Milli Q water was added and the falcon tube was capped under sterile conditions. The sample was subsequently placed in a hot water bath at 55 °C for 1 hour to prevent growth of fast-growing gram-negative bacteria and fungi. The heated soil sample was allowed to cool and then placed in a clean bench which had been sterilized with 70% ethanol followed by exposure to ultraviolet (UV) light for an hour. About 10 mL of sterilized tap water was added to sample and dilutions of 10^{-1}, 10^{-2} and 10^{-3} concentrations were prepared. This was done by pipetting 1 mL of the stock 10 mL suspension into a new sterile 50 mL falcon tube and subsequently diluting with 9 mL of sterilized tap water after which it was shaken thoroughly to afford the 10^{-1} dilution. A 1 mL aliquot of the 10^{-1} suspension was used to prepare the subsequent serial dilutions. Pre-sterilized inoculation loops were used to smear evenly about 5 µL of the different concentrations of the soil sample on pre-modified ISP2 agar plates at pH 5.5 supplemented with 25 µg/mL each of nystatin and nalidixic acid antibiotics. The master plates were parafilmed and incubated for 7 days at 28 °C to allow the growth of bacteria. After the 7-day period, the agar plates were taken out of the incubator and observed in a clean bench. Single colonies with different phenotypes were re-plated on new agar plates using sterilized inoculation loops in the clean bench to obtain pure strains. Parent or master plates that had fungal contamination or did not have single individual colonies to pick were discarded to prevent contamination of the lot. The plates were sealed using parafilm and put in the incubator to allow for continued bacteria growth. Pure strains that had been transferred onto new agar plates were also parafilmed and placed in the incubator for periodic observation. Pure strains were obtained by successively sub-culturing all colonies originally sub-cultured from the parent or master plates. A pure strain of *Bacillus* sp. DE2B was obtained through this process and its whole DNA submitted to the Department of Biochemistry, Sanger Sequencing, University of Cambridge, UK for whole genome sequencing.

Identification of strain DE2B

Identification of the closest phylogenetic neighbor and calculations of pairwise 16S rRNA gene sequence similarities were achieved with the EzBioCloud web service by Yoon et al., 2017 [2]. Multiple sequence alignments were obtained using the CLUSTAL W application by Thompson, Higgins and Gibson, 1994 [3] and phylogenetic analysis was performed using the MEGA v.7.0 software package by Kumar et. al. in 2016 [4]. Evolutionary distance calculations were obtained using the Jukes–Cantor model [5] while Neighbor-joining was achieved using Saitou and Nei 1987 [6]. Maximum-parsimony, Felsenstein, 1983 [7] and Maximum-likelihood, Felsenstein, 1981 [8] methods were used to infer the phylogenetic tree. The resultant tree topologies were assessed by bootstrap analyses, Felsenstein, 1985 [9] based on 1000 re-samplings of the datasets. The relative analysis of the 16S rRNA gene sequence of strain DE2B, revealed its phylogenetic association to the genus Bacillus, particularly, members of the *Bacillus cereus* group sharing over 98.05% similarity with the known species of this group, Figure S3. *Bacillus* sp. strain DE2B showed highest 16S rRNA gene sequence resemblances with the strains *B. cereus* (99.93%), *B. albus* (99.86%), *B. wiedmannii* (99.79%), and *B. proteolyticus* (99.72%), and other species of the *B. cereus* group.

Culture and fermentation of strain DE2B

Bacillus sp. strain DE2B was grown on ISP2 agar plates made up of 10 g of malt extract, 4 g each of yeast extract and D-glucose and 15 g of agar at pH 5.5 with incubation temperature 28 °C for 72 hours. Subsequently, a small-scale culture was prepared by inoculating 100 mL of a TSBY fermentation media broth composed of 17 g tryptone, 3 g of phytone, 5 g of sodium chloride and 2.5 g of each disodium phosphate and glucose in distilled water in a 250 mL Erlenmeyer flask plugged with non-absorbent cotton wool and autoclaved with spores of the strain DE2B. This small-scale culture was incubated for 14 days at 28 °C, 220 rpm and 25 g/L of autoclaved HP-20 resin added under sterile conditions.

For large scale fermentation cultures, an inoculum was prepared by placing 50 mL of TSBY fermentation media in a 250 mL Erlenmeyer flask plugged with non-absorbent cotton wool and autoclaved and subsequently inoculating this with spores of the strain DE2B followed by incubation for three days at 28 °C with 220 rpm. The inoculum was used to inoculate nine 1 L

Erlenmeyer flasks each containing 200 mL of TSBY media, plugged with non-absorbent cotton wool and previously sterilized. These flasks were then cultured for 14 days at 28 °C, 220 rpm. Exactly 24 hours prior to the end of the culture periods, 50 g/L of autoclaved HP-20 resin was added to each flask under sterile conditions and returned to the incubator.

Extraction and purification procedures

The culture broths of *Bacillus* sp. strain DE2B (1.8 L) were filtered through glass wool in a Büchner funnel under suction to separate the mycelia and supernatant. The mycelia with the HP-20 resin was placed in a 1 L Erlenmeyer flask and extracted alternatively and sequentially with CH_2Cl_2 and MeOH. The supernatant was extracted with EtOAc once. All extracts were combined and dried under vacuum to obtain a total crude extract (1.48 g). A modified Kupchan's solvent partitioning [10] of the total crude extract gave four fractions: FH (217 mg), FD (554 mg), FM (167 mg), and WB (275 mg) with FD being the fraction of interest. Size exclusion chromatography of the FD fraction using Sephadex LH-20 (Sigma Aldrich, Munich, Germany) as a stationary phase yielded seven fractions labelled FD-SFA-G. ^{1}H NMR and microchemical screening using ninhydrin reagent for all Sephadex LH-20 fractions showed that FD-SFB (154 mg) contained the compounds of interest. The fraction FD-SFB was further subjected to purification on a reversed phase semi-preparative HPLC using a Phenomenex Luna reversed-phase C-18 column (5 µm, 100 Å 250 × 6.10 mm, L × i.d.) and a gradient elution with solvent A = 80/20% (milliQ-H_2O/CH_3CN) and B = 100% (CH_3CN) at (100% A to 100% B in 20 min and hold for 40 min) and a column flow rate set to 1.5 mL/min. Compound **1** eluted at retention time 26.0–30.0 min with mass 7.68 mg. Compound **1** was observed to be an odorless white amorphous powder when dried under vacuum.

Physical data for compound 1

Odorless amorphous white powder; $[\alpha]^{24}_{D}$ (*c* 0.1, MeOH) −30.0, IR (neat) ν_{max} 3298, 2958, 2927, 2852, 1643, 1542, 1208 cm^{-1}; UV (CH_3CN) λ_{max} (logε) = 220 (2.77); HR-ESI-MSMS (*m/z*): 1008.655 [M+H]$^+$ (calculated for $C_{51}H_{90}N_7O_{13}^+$, 1008.659); ^{1}H, ^{13}C, HSQC, COSY, HMBC, HSQC-TOCSY and NOESY NMR data are shown in Table S1. Mass spectrometry data is detailed in figure S17

Determination of absolute configuration for amino acid residues in the structure of compound 1

About 0.2 mg of compound **1** was hydrolyzed in 0.4 mL of 6 N HCl for 24 hours at 110 °C. The reaction mixture was cooled, and the solvent evaporated under vacuum. Residual HCl was washed off completely by the addition of 0.4 mL of water followed by the removal of all solvent. The washing with water was repeated three times in order to ensure that all residual HCl was removed after the hydrolysis process was completed. The hydrolysate was subsequently dried completely under N_2 for 24 hours and the residue dissolved in 50 µL of H_2O. 100 µL of 25% (v/v) Et_3N and 100 µL of 1% L-FDLA (Marfey's reagent) in acetone were added either to 50 µL of a 50 mM standard aqueous amino acid solution or alternatively, the hydrolysate. The reaction mixtures were incubated for 1 h at 40 °C, with regular mixing. The reaction mixtures when cooled to room temperature were quenched by the addition of 20 µL of 2N HCl [11]. Samples were diluted to a 1 mL volume with MeOH and 50 µL aliquots of each sample were analyzed by HPLC using a Phenomenex C_{18} column (5 µm, 100 Å, 4.6 mm × 250 mm), eluted with a solvent gradient of 100% solvent A (H_2O/CH_3OH 80:20) to 100% solvent B (CH_3OH) at 30 minutes at a flow rate of 1 mL min^{-1}. The comparison of the retention times and UV profiles of derivatized hydrolyzed amino acids with the retention times of derivatized D- and L-amino acid standards revealed an L configuration for all Glu, Val and Leu amino acid residues except for Asp which was of the D configuration. Interestingly, the yields for the Marfey reagent reaction with D-Asp acid had greater yields than same reactions with L-Glu, L-Val and L-Leu. A table of the HPLC retention times for the different amino acids present in both the hydrolysate and standards along with the UV profiles of Marfey derivatized residues are shown in Table S3 and Figure S9–S13.

Bioassay reagents

RPMI-1640, IMDM, M-199, HEPES, YI-S, foetal bovine serum (FBS), adult bovine serum (ABS), gentamycin, penicillin-streptomycin-L-glutamine (PSG), 2-[4-(2-hydroxyethyl) piperazin-1-yl] ethanesulfonic acid (HEPES), ampicillin, sumarin, amphotericin B, alamar dye, dimethyl sulfoxide (DMSO), sodium citrate, adenine, sodium bicarbonate ($NaHCO_3$), AlbuMax II, sodium chloride (NaCl), sodium phosphate dibasic (Na_2HPO_4), potassium chloride (KCl),

sodium phosphate monobasic (KH_2PO_4), sodium hydroxide (NaOH), and sodium bicarbonate ($NaHCO_3$) were purchased from Sigma-Aldrich, USA.

Preparation of compound for bioassay

A stock solution of 10 mM concentration of compound **1** was prepared by first drying the compound using nitrogen gas and weighing on a mass balance (AND GH-120, A and D Company Limited, Tokyo, Japan) to determine the weight before dissolving in an appropriate volume of dimethyl sulfoxide (DMSO) to obtain the 10 mM concentration. Subsequently, the solution was vortexed (MSI Minishaker, IKA Company, Osaka, Japan) and filter sterilized into vials through 0.45 µm millipore filters under sterile conditions. The solution was then stored at −20 °C until used.

Cell culture

The GUTat 3.1 strain of the bloodstream form of *T. brucei brucei* parasites was used in this study. Parasites were cultured in vitro according to the conditions established previously by Yabu et al. in 1998 [12]. Parasites were used when they reached a confluent concentration of 1×10^6 parasites/mL. Estimation of parasitemia was done with the Neubauer counting chamber. Parasites were diluted to a concentration of 3×10^5 parasites/mL with IMDM medium and used for the drug assay.

The log-phase promastigotes of *L. donovani* (D10) and *L. major* (NR48815) were cultured in M-119 growth medium with a working concentration of 6×10^6 cells/mL. Parasites were used when they reached a confluent concentration of 1×10^6 parasites/mL. Estimation of parasitemia was done with the Neubauer counting chamber. Parasites were diluted to a concentration of 3×10^5 parasites/mL with M199 medium and used for the drug assay [13].

Mouse macrophages (RAW 264.7 cell lines) were cultivated in vitro to the log-phase using Dulbecco's modified eagle medium (DMEM, Thermo Fisher Scientific) with 10% FBS at 5% CO_2 and 37 °C [14].

In vitro viability test for trypanosomes and macrophages

The viability of the treated or untreated trypanosome parasites were ascertained by the Alamar Blue assay test in a manner similar to a procedure from [13]. The assay was carried out in a 96-well plate through the instructions of the manufacturer with slight modifications. About 1.5×10^4 parasites were seeded with varied concentrations of the compound **1** ranging from 0 µM to 100 µM. Final concentrations of DMSO were maintained at 0.1%, respectively. After incubation of parasites with or without the compound for 24 hours at 37 °C in 5% CO_2, 10% Alamar Blue dye was added, and the parasites were incubated for another 24 hours in darkness. A Tecan Sunrise Wako spectrophotometer was used to read the absorbance at 540 nm for the plate after 48 hours. Suramin was used as the control.

For normal macrophages RAW 264.7, cell lines were plated at a density of 3.0×10^5 cells/mL for 48 hours to allow for sufficient adherence to plates before adding the compound to the cells in a two-fold dilution and subsequent incubation for another 24 hours.

In vitro viability test for Leishmania parasites

As previously described in [14], the Alamar Blue assay was carried out on treated and untreated Leishmania parasites to ascertain their viability. The assay was performed in a 96-well plate following the manufacturer's instructions, with modification. About 3×10^5 parasites were seeded with varied concentrations of the compound ranging from 0 µM to 100 µM. Final concentrations of DMSO were kept at 0.1%. After incubation of parasites with or without the compound for 24 hours at 28 °C, 10% Alamar Blue dye was added, and the parasites were incubated for another 24 hours in darkness. After a total of 48 hours, the plate was read for absorbance at 540 nm using a Tecan Sunrise Wako spectrophotometer, AUSTRIA GmbH (Salzburg, Austria). The trend curve was drawn to obtain a 50% inhibitory concentration (IC_{50}) for the compound.

In vitro viability test for laboratory microbes

In a manner similar to [1], nine different standard bacteria strains, 7 Gram negative, *Eschericha coli* (ATCC 25922), *Salmonella paratyphi ß* (9150), *Shigella flexneri* (ATCC 12022), *Shigella dysenteriae* (13313), *Salmonella typhimurium* (14028), *Salmonella enteritidis* (ATCC 13076)

and *Bacillus cereus* (ATCC 14579) and 2 Gram-positive, *Staphylococcus aureus* (ATCC 29213), and *Staphylococcus epidermidis* (ATCC 12228) were used in this study. Each stock standard bacteria strain was incubated overnight at 37 °C on a Mueller–Hinton agar (Park Scientific Limited) plate before the antimicrobial assay. Three individual colonies from the bacteria plate were selected, transferred into media and incubated at 37 °C overnight for the bacteria to reach the log-phase of growth. The log-phase bacteria were diluted with sterile saline to achieve a turbidity of 0.5 McFarland standards which was approximated to be concentration 2×10^8 CFU/ml. The bacteria were then diluted to the working concentration, which varied between bacteria. Log-phase of bacteria at a concentration range of 1×10^2 to 1×10^6 CFU/mL were incubated with different concentrations of the compound (100–0 μM) and 10% Alamar Blue® reagent at 37 °C for 6–8 hours. Absorbance was read at wavelengths of 540 nm for the samples and 595 nm for the reference standard using a TECAN Sunrise Wako spectrophotometer, Austria GmbH. IC_{50} values of compounds were calculated by linear regression. Ampicillin was used as positive control except for *Salmonella paratyphi ß* (9150) where amphotericin B was used as the control.

Supplementary tables

Table S 1. 1D and 2D NMR spectroscopic data for compound 1 acquired in DMSO-d_6.

**Residue**	**#**	**δ_C, mult**	**δ_H, mult (*J* Hz)**	**^{1}H-^{1}H COSY**	**HMBC**	**HSQC-TOCSY**	**NOESY**
**glutamic acid**	1	171.8, C					
	2	51.9, CH	4.19, ov.	1NH, 3	1	1NH, 3, 4	1NH, 2NH
	3	27.3, CH_2	1.91, ov.<br>1.77, ov.		1, 2, 4	1NH, 4	1NH, 4
	4	29.9, CH_2	2.22, t (7.9)	3	2, 3 , 5	2, 3	1NH, 3
	5	174.0, C					
	1NH		7.82, d (7.2)	2	37		2,3,4,38,39, 40
	OH		12.24, s				
**valine-1**	6	170.6, C					

	7	58.2, CH	4.08, dd (8.8, 6.0)	2NH	6	2NH, 8, 9, 10	3NH
	8	30.5, CH	1.96, ov.		6, 7		9,10
	9,10	17.9, CH_3	0.75, d (6.8)		7	2NH, 8	8
	2NH		7.77, ov.		1		2
**aspartic acid**	11	169.8, C					
	12	49.6, CH	4.53, ov.	3NH, 13	13	3NH, 13	3NH, 13
	13	35.9, CH_2	2.70, dd (7.2, 18)<br>2.59, dd (9.5, 18)		11, 12, 14	3NH, 12	3NH, 4NH, 12
	14	171.3, C					
	3NH		8.17, m		6		7, 12, 13
	OH		12.24, s				
**leucine-1**	15	171.8, C					
	16	50.6, CH	4.52, ov.	4NH	15	4NH, 17, 19, 20	4NH, 5NH, 17
	17	41.6, CH_2	1.42, m	16	15	4NH, 16, 19, 20	16
	18	24.1, CH	1.53, ov.			19, 20	
	19,20	22.9, CH_3	0.86, ov.		18		
	4NH		7.68, d (8.6)		11		13,16
**valine-2**	21	172.2, C					
	22	57.2, CH	4.18, ov.	5NH	21	5NH, 23	6NH
	23	29.5, CH	2.07, h (6.0)		21, 22, 25		5NH
	24	19.0, CH_3	0.84, ov.	23	22	23	
	25	18.9, CH_3	0.84, ov.	23	22	5NH, 23	6NH
	5NH		8.26, ov.		15		16, 23, 24
**leucine-2**	26	171.6, C					
	27	50.4, CH	4.44, ov.		26	28, 30, 31	7NH
	28	41.6, CH_2	1.42, ov.	27	26	27, 30, 31	27

	29	24.1, CH	1.53, ov.			30, 31	
	30, 31	23.0, CH_3	0.86, ov.		29		
	6NH		8.30, ov.		21		22, 25
**valine-3**	32	170.6, C					
	33	58.2, CH	4.08, dd (8.8, 6.0)		32	7NH, 34, 35, 36	7NH
	34	30.5, CH	1.96, ov.		32, 33		7NH, 35,36
	35, 36	17.9, CH_3	0.75, d (6.8)		33	7NH, 34	34
	7NH		8.26, ov.				27, 33, 34
**fatty acid**	37	169.4, C					
	38	40.4, CH_2	2.41, ov. 2.38, ov.	39	39	39	1NH, 39, 40
	39	71.6, CH	5.00, m	38	37	38, 45, 46, 42, 43, 44, 41, 40, 48	1NH, 38, 40, 41, 42,
	40	33.2, CH_2	1.55, ov.			39, 38, 45, 46, 43, 44 41, 42	1NH, 38, 39
	41	28.5, CH_2	1.21, ov.				
	42	28.8, CH_2	1.21, ov.			39,48, 40	39
	43	28.9, CH_2	1.21, ov.				
	44	28.9, CH_2	1.21, ov.				
	45	28.9, CH_2	1.21, ov.		47		
	46	28.9, CH_2	1.21, ov.		47		
	47	26.7, CH_2	1.22, ov.		45, 46	41, 42, 43, 44, 45,46	
	48	39.7, CH_2	1.53, ov.		50	50, 51	
	49	27.3, CH	1.49, ov.		48		
	50, 51	23.0, CH_3	0.86, ov.		49		
	1NH				37		

Table S2. 1D and 2D NMR spectroscopic data for compound 1 acquired in $CDCl_3$.

Residue	#	δc , mult	δ_H, mult (J Hz)	^{1}H- ^{1}H COSY	HMBC	HSQC-TOCSY
**valine-1**	7	56.7, CH	4.39, s	8		2NH, 9, 10
	8	31.7, CH	2.16, ov.	7		9, 10
	9	19.2, CH_3	0.82, ov.		7	
	10	19.2, CH_3	0.88, ov.		7, 9	
	2NH					
**aspartic acid**	12	49.4, CH	4.92, s			3NH, 13
	13	36.5, CH_2	3.08, m<br>2.68, m			12, 3NH
	3NH					
**leucine-1**	16	51.4, CH	4.72, s	4NH, 18		4NH, 18, 19, 20
	17	40.5, CH_2	1.72, m<br>1.59, m		16	
	18	24.7, CH	1.65, ov.	16	19, 20	16, 19, 20
	19	22.7, CH_3	0.95, ov.<br>0.93, ov.<br>0.85, ov.		18, 20	16, 18
	20	22.7, CH_3	0.95, ov.<br>0.93, ov.<br>0.85, ov.		18, 19	16, 18
	4NH					
**valine-2**	22	56.7, CH	4.39, s	23		5NH, 24, 25
	23	31.7, CH	2.16, ov.	22		
	24	19.4, CH_3	0.91, ov.		22	
	25	19.4, CH_3	0.91, ov.		22	
	5NH					
**leucine-2**	27	53.4, CH	4.72, s	28		28, 30, 31
	28	40.5, CH_2	1.72, m<br>1.59, m	27	30, 31	30, 31, 27
	29	24.7, CH	1.65, ov.		30, 31	27
	30	22.7, CH_3	0.95, ov.<br>0.93, ov.<br>0.85, ov.			27
	31	22.7, CH_3	0.95, ov.<br>0.93, ov.<br>0.85, ov.			27
	6NH					

**valine-3**	33	61.8, CH	3.95, s	7NH, 34		7NH, 34, 35, 36
	34	29.3, CH	2.19, ov.	33, 35, 36		33
	35	19.3, CH_3	1.04, ov.		33	34
	36	19.3, CH_3	0.98, ov.		33	33, 34
	7NH					
**fatty acid**	38	39.6, CH_2	2.81, s<br>2.34, ov.	39		40, 39
	39	73.0, CH	5.08, s	40, 38		38, 40), 47
	40	33.7, CH_2	1.92, ov.<br>1.61, ov.	39, 38		39, 38
	41	29.3, CH_2	1.25, ov.			
	42	29.5, CH_2	1.25, ov.			39
	43	29.5, CH_2	1.25, ov.			39
	44	29.6, CH_2	1.22, s			47
	45	29.6, CH_2	1.22, s		48	47
	46	29.7, CH_2	1.25, ov.		48	
	47	25.6, CH_2	1.29, ov.		48	39
	48	39.0, CH_2	1.13, m	49	49	44, 45, 49
	49	28.0, CH	1.50, hept	48	48, 50, 51	
	50	22.7, CH_3	0.95, ov.<br>0.93, ov.<br>0.85, ov.		48, 51	48, 49
	51	22.7, CH_3	0.95, ov.<br>0.93, ov.<br>0.85, ov.		48, 50)	48, 49

Table S3. Reverse Phase HPLC retention times (RT) for the standard amino acids (SAA) and the amino acids present in compound 1 (AAC1).

**SAA**	R_T (min)	**AAC1**	R_T (min)
L-Asp	4.306	-	-
D-Asp	7.273	D-Asp	7.873
L-Glu	7.023	L-Glu	7.873
L-Val	11.330	L-Val	11.826
L-Leu	14.526	L-Leu	14.441

Supplementary figures

Figure S1: Pure *Bacillus* sp. strain DE2B growing on ISP2 agar plates at pH 5.5 and 28 °C incubation temperature.

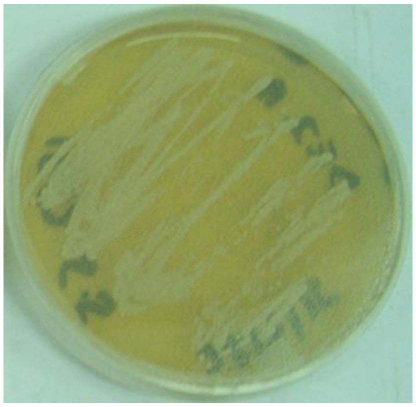

Figure S2: Phylogenetic tree of Bacillus sp. DE2B.

* Neighbour-joining phylogenetic tree based on 16S rRNA gene sequences showing the position of strain *Bacillus* sp. DE2B among species of the *Bacillus cereus* group. *B. subtilis* ATCC 6051T was used as an out-group. Bootstrap values (based on 1000 replicates) greater than 50 % are shown at nodes. Bar, 0.005 substitutions per nucleotide position.

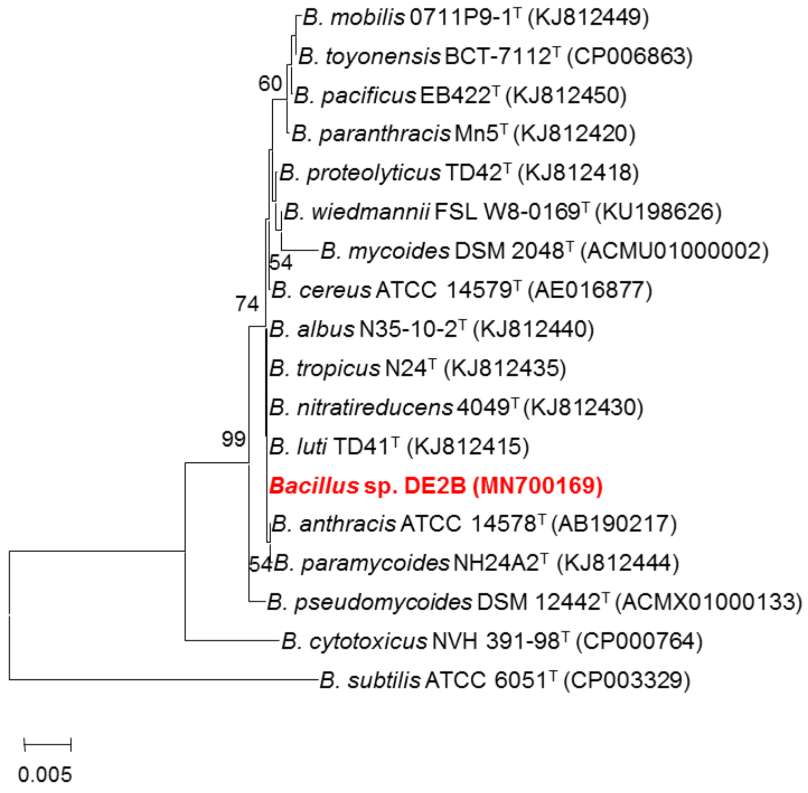

Figure S3: Modified Kupchan solvent partitioning of the crude extract of Bacillus sp. DE2B gives FH, FD, FM, and WB fractions.

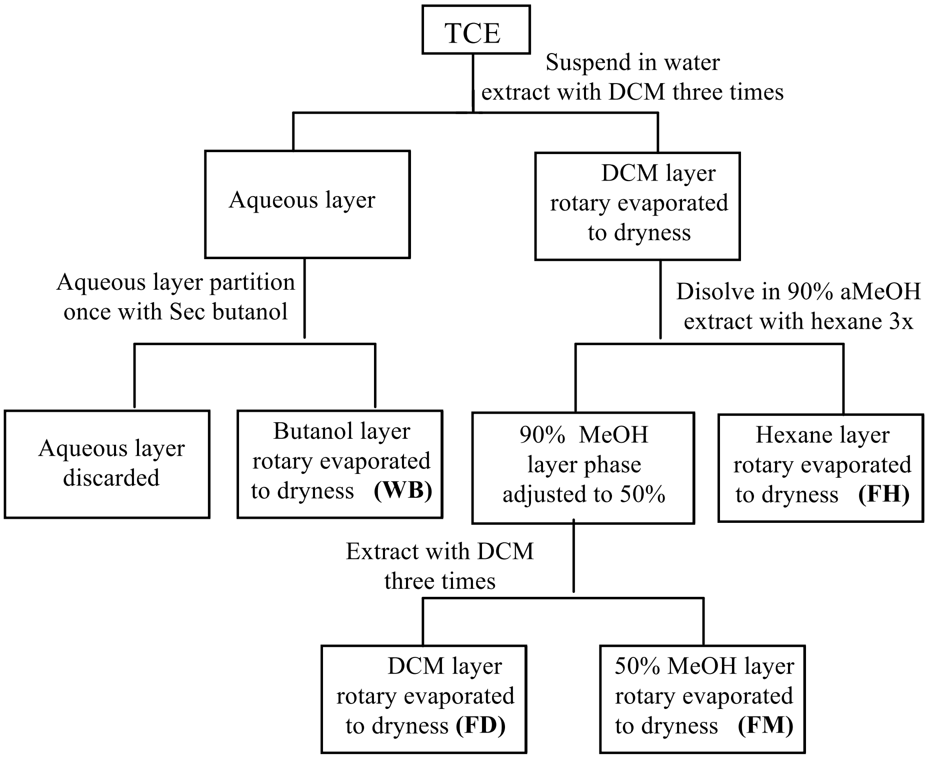

Figure S4: Sephadex LH-20 chromatography of FD fraction followed by reversed phase semi-preparative HPLC gives pure compound **1**.

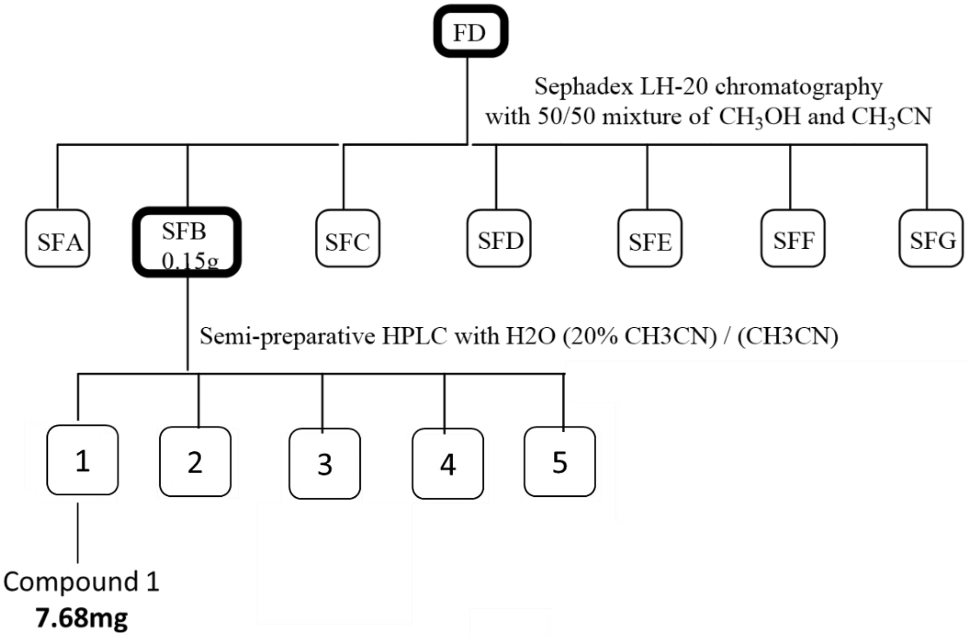

Figure S5: First HPLC chromatogram for FD-SFB.

***First HPLC chromatogram for FD-SFB produces the five-member homologous series of cyclic lipopeptides **1-5**. Compound **1** is collected and re-injected on HPLC to further purify it for full structure elucidation.

Stationary Phase-C18 Reversed Phase Column; Mobile Phase-Gradient Mixtures of Solvent A = 80% H_2O/20% CH_3CN and Solvent B = 100% CH_3CN; Runtime Flow Rate 60 Minutes at 1.5 ml/min; Detetor-PDA detection at Wavelength 200-400nm.

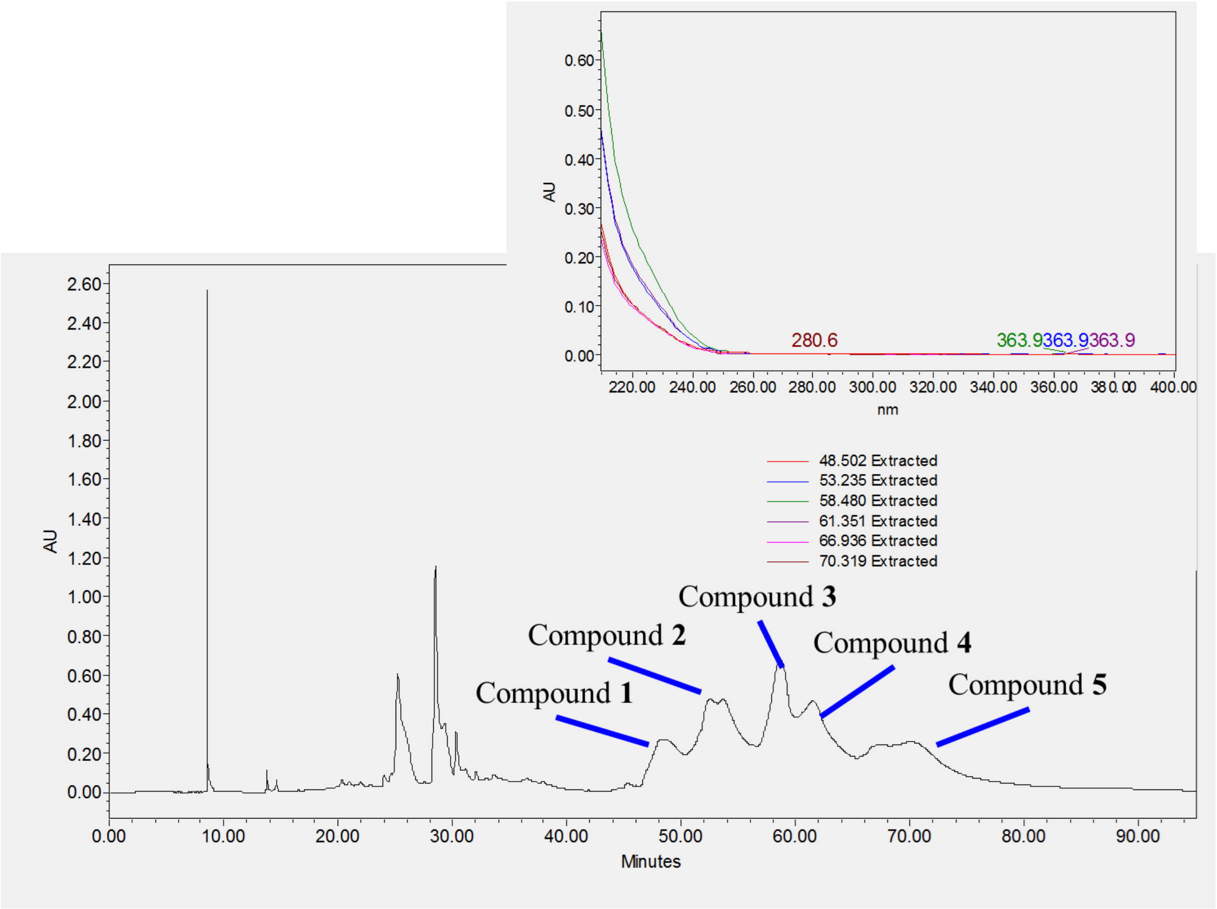

Figure S6: Repeated HPLC chromatogram for compound 1 collected from the first HPLC in Figure S6.

*Stationary Phase-C18 Reversed Phase Column; Mobile Phase-Gradient Mixtures of Solvent A = 1% CH_2O_2/79% H_2O/20% CH_3CN and Solvent B = 100% CH_3CN; Runtime Flow Rate 60 Minutes at 1.5 ml/min; Detetor-PDA detection at Wavelength 200-400nm.

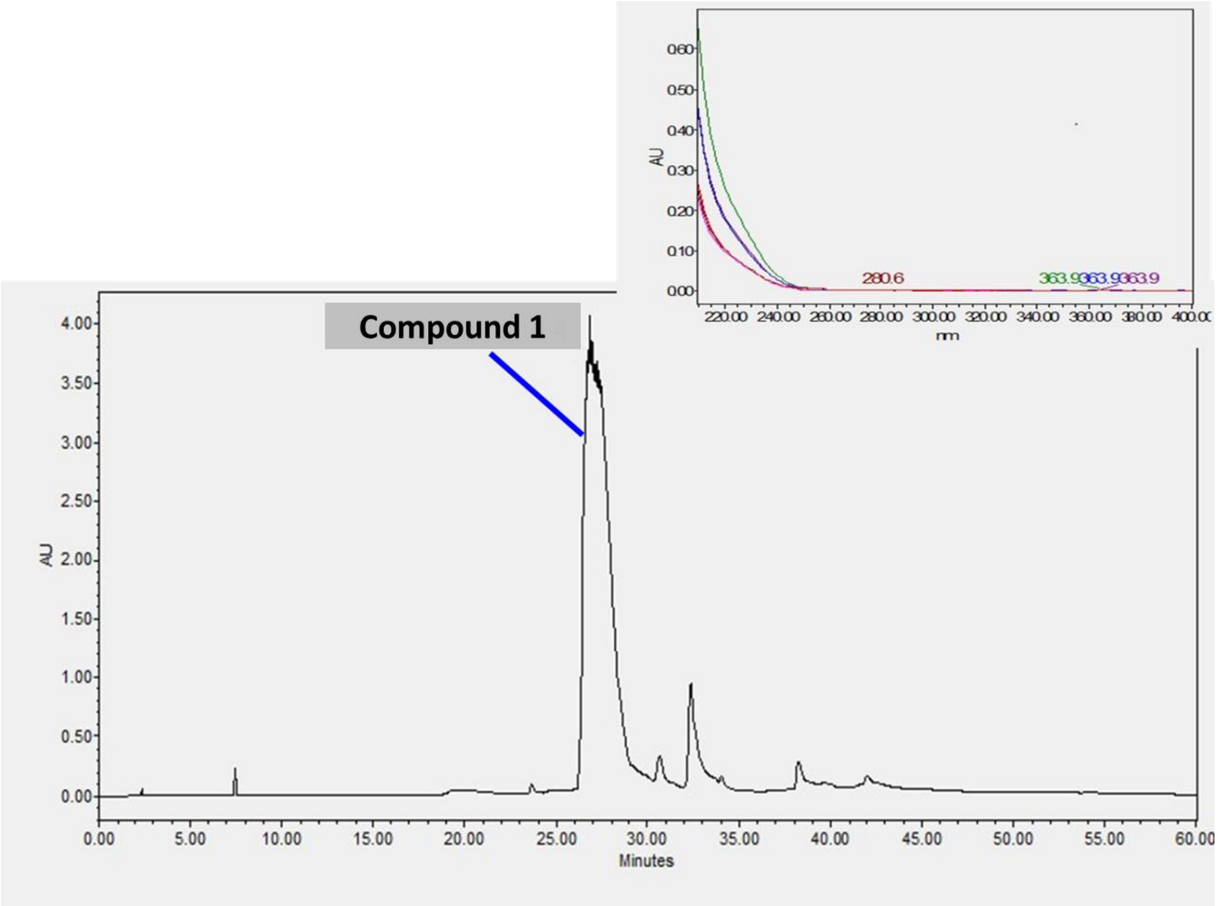

Figure S7: Infrared spectrum of compound **1.**

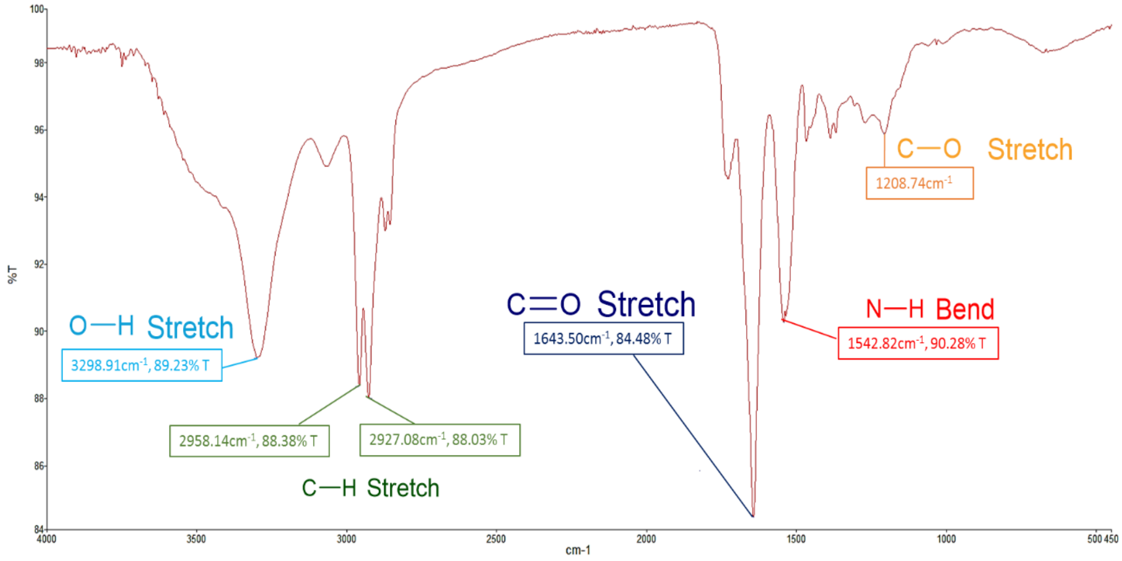

Figure S8: Reversed phase HPLC chromatogram for Marfey derivatised amino acid residues from compound **1**.

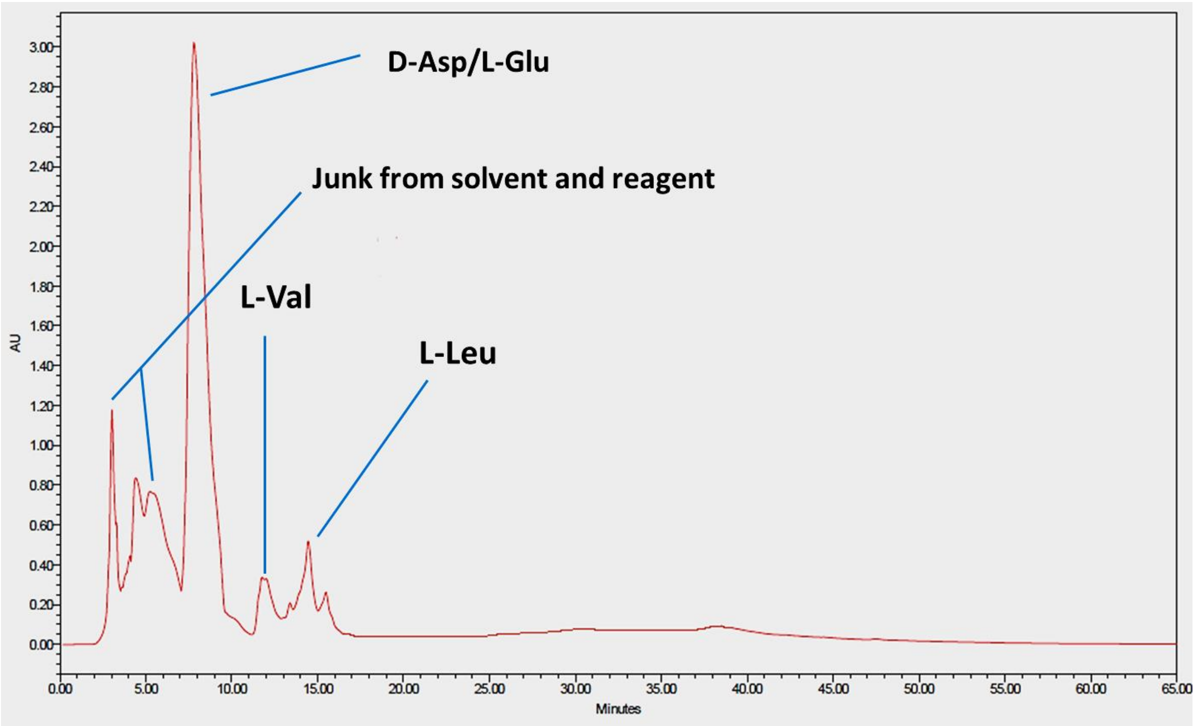

Figure S9: Reversed phase HPLC chromatogram and UV profile of Marfey derivative for L-Glu residue.

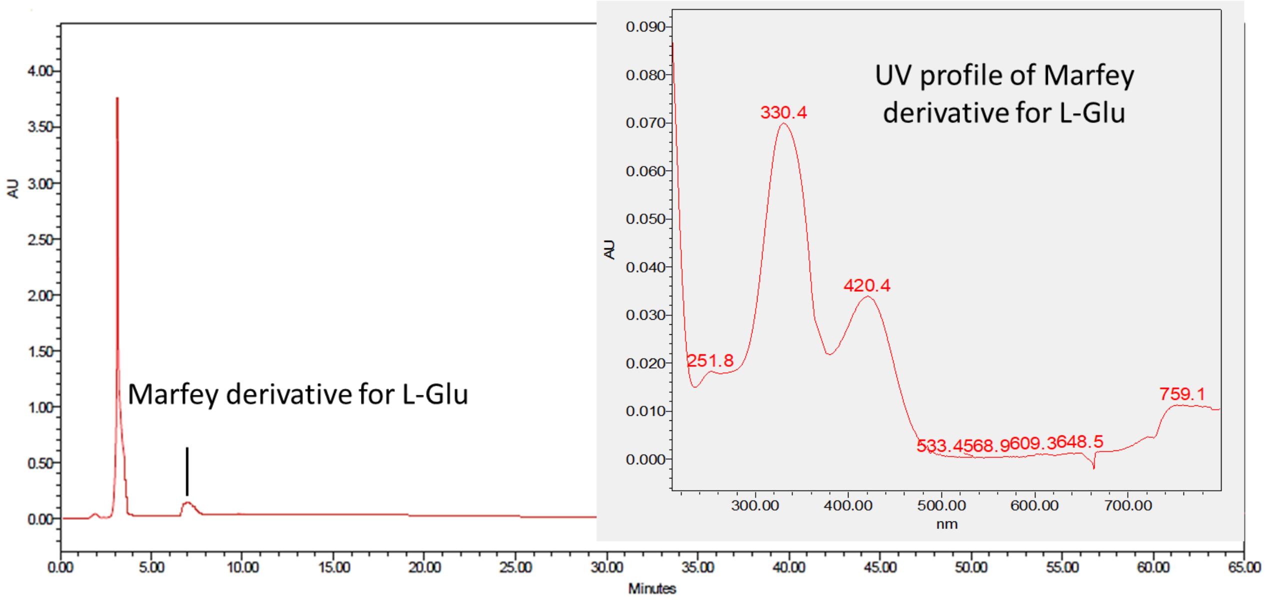

Figure S10: Reversed phase HPLC chromatogram and UV profile of Marfey derivative for L-Val residue.

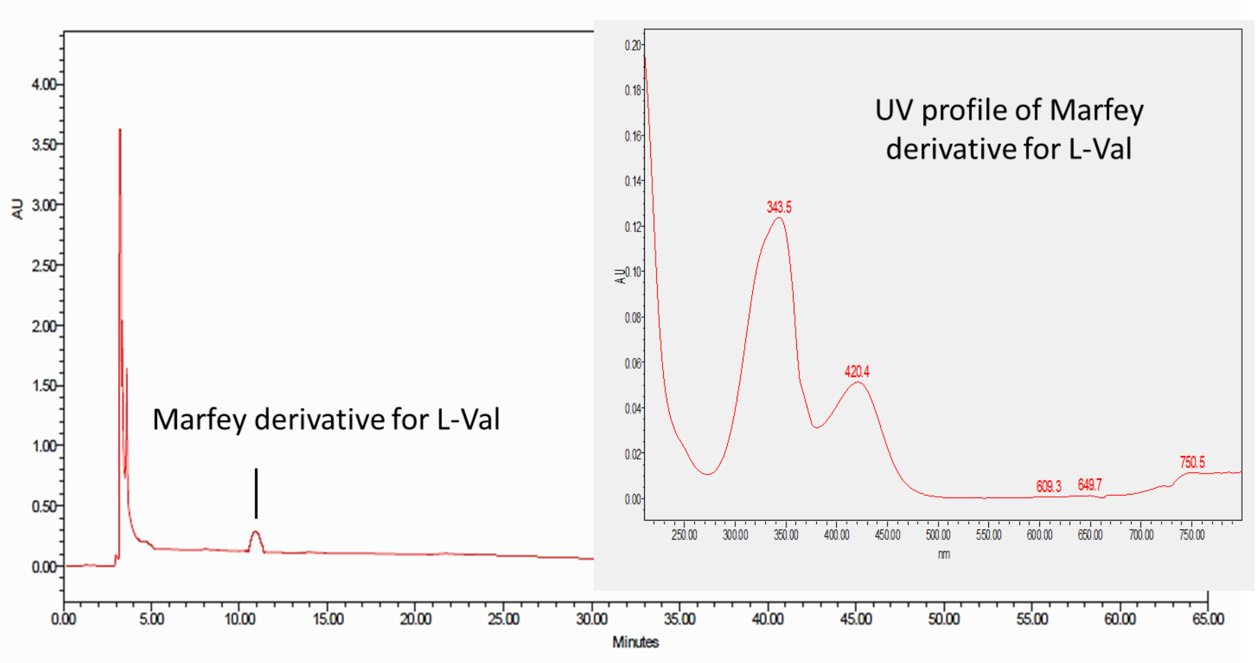

Figure S11: Reversed phase HPLC chromatogram and UV profile of Marfey derivative for L-Leu residue.

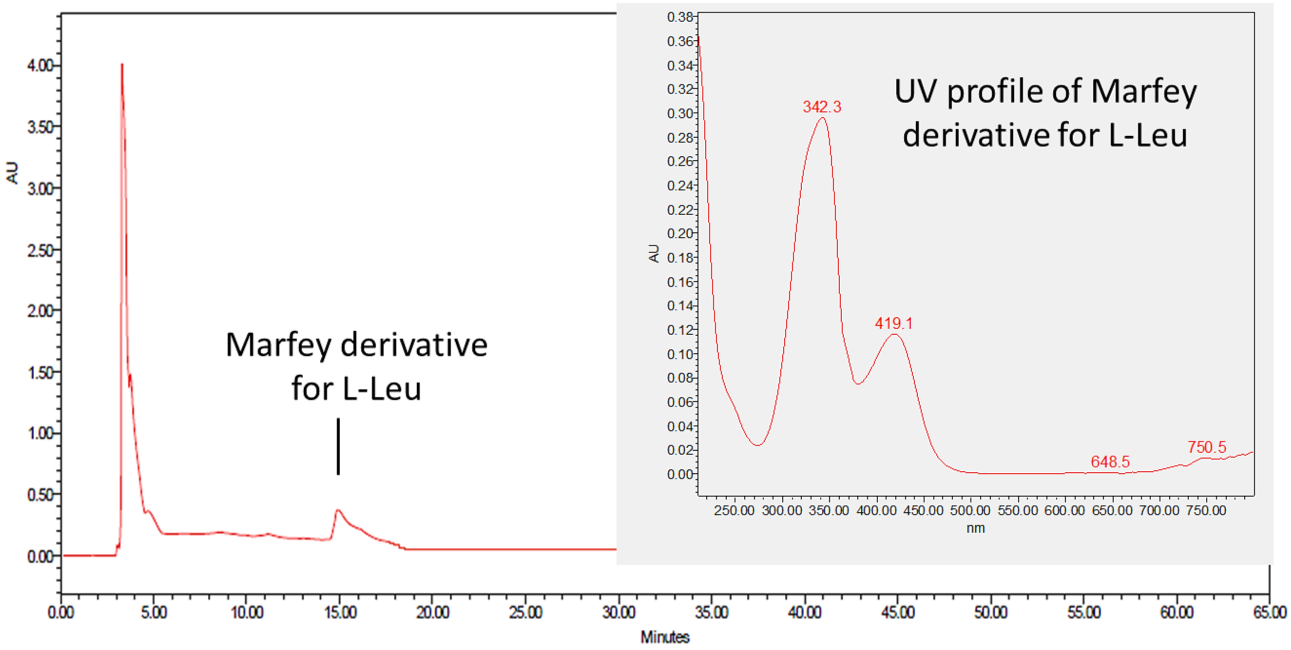

Figure S12: Reversed phase HPLC chromatogram and UV profile of Marfey derivative for D-Asp residue.

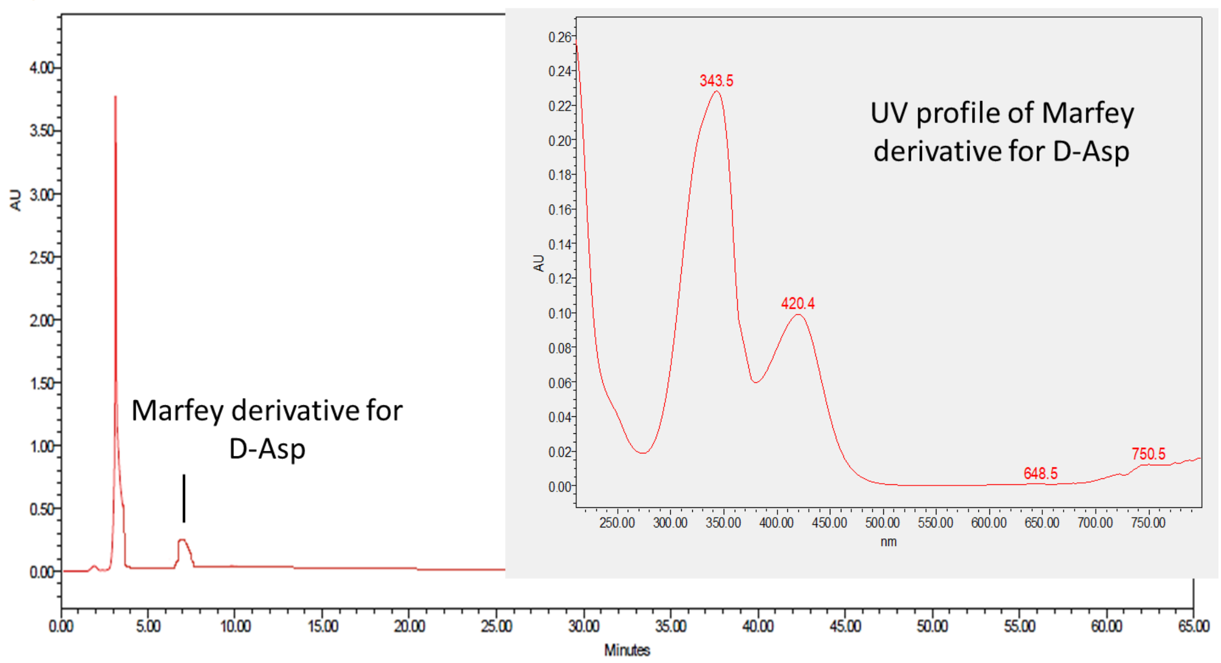

Figure S13: The IC50 plot for the observed antileishmania activity of compound **1** using Leishmania donovani (Laveran and Mesnil) Ross (D10).

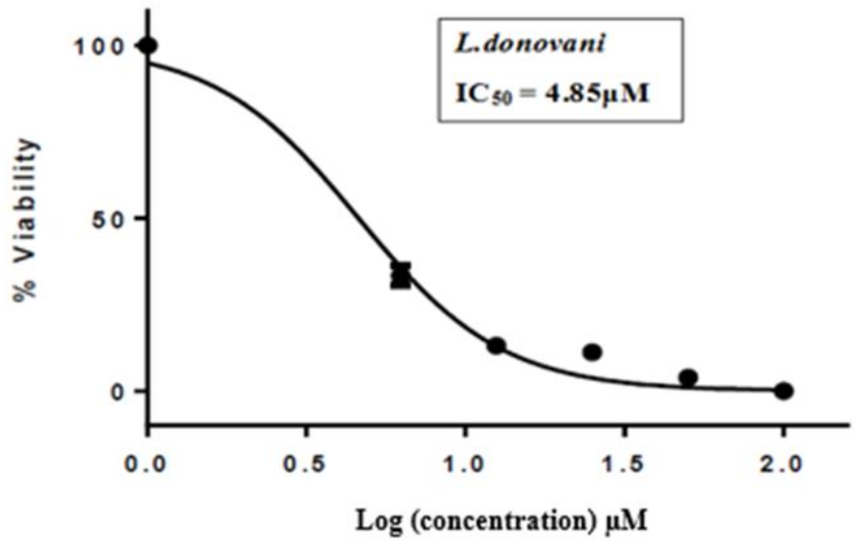

Figure S14: The IC50 plot for the observed antitrypanosomal activity of compound **1** using Trypanosoma brucei subsp. brucei strain GUTat 3.1.

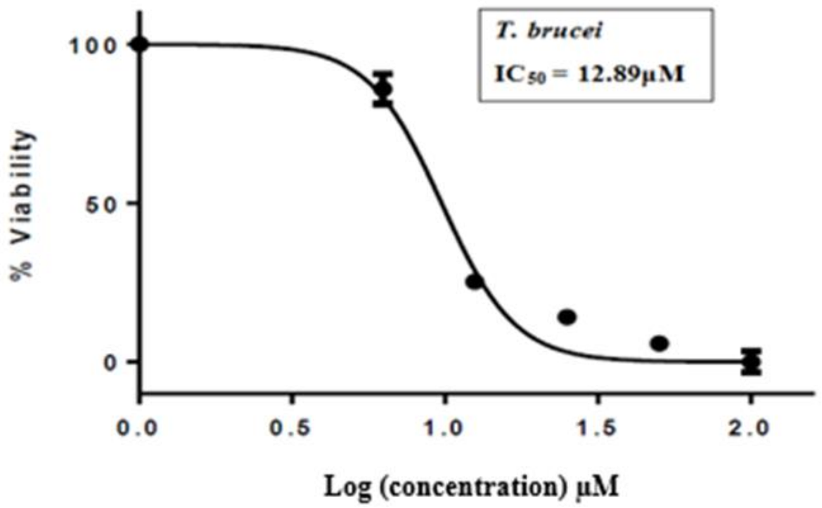

Figure S15: IC_{50} plot showing cytotoxicity test of compound **1** using Raw 264.7 cell lines (macrophages).

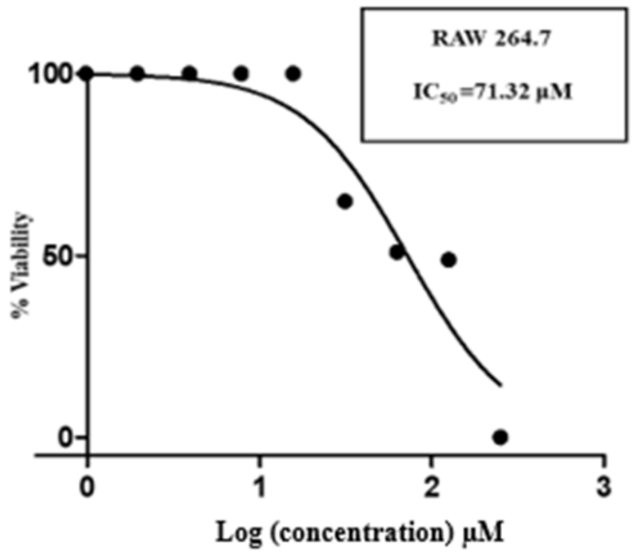

Figure S16: HR-ESI-LC-MSMS data showing fragmentation pattern for compound **1**.

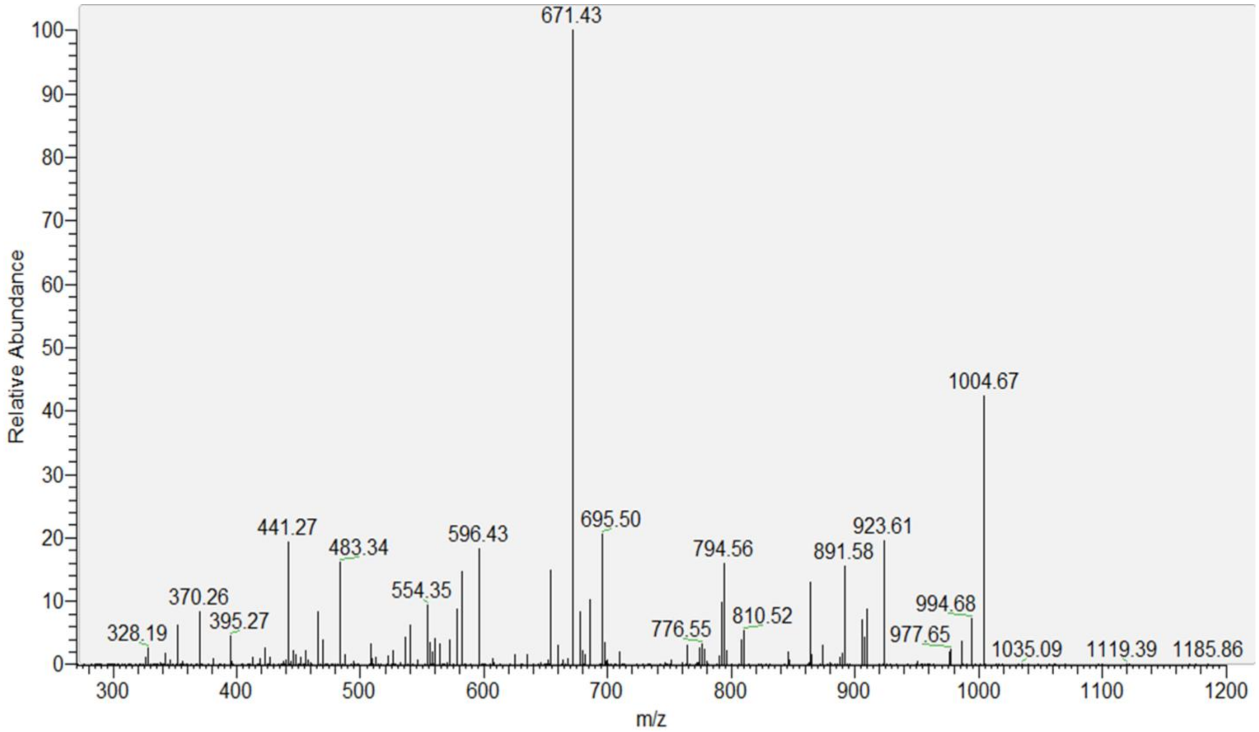

.

Figure S17: HR-ESI-LC-MSMS data with sequence tag information for compound **1**.

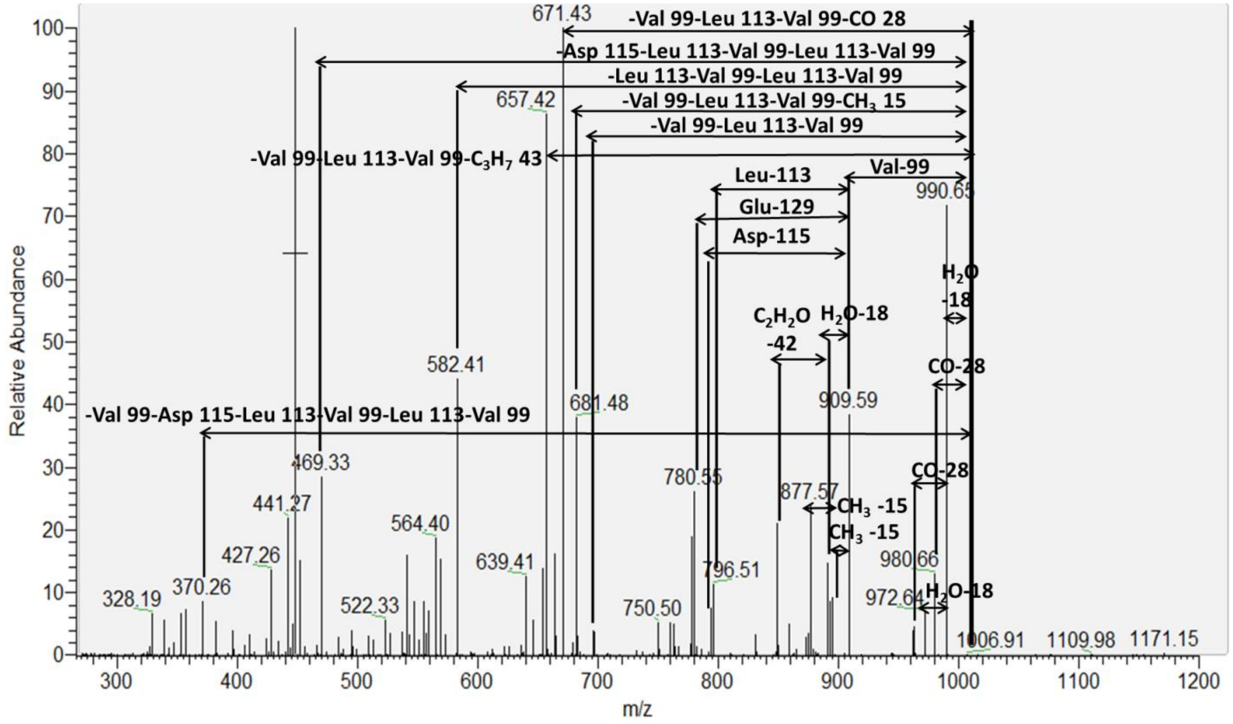

Figure S18: 500 MHz ^{1}H NMR spectrum of compound **1** in DMSO-d_6.

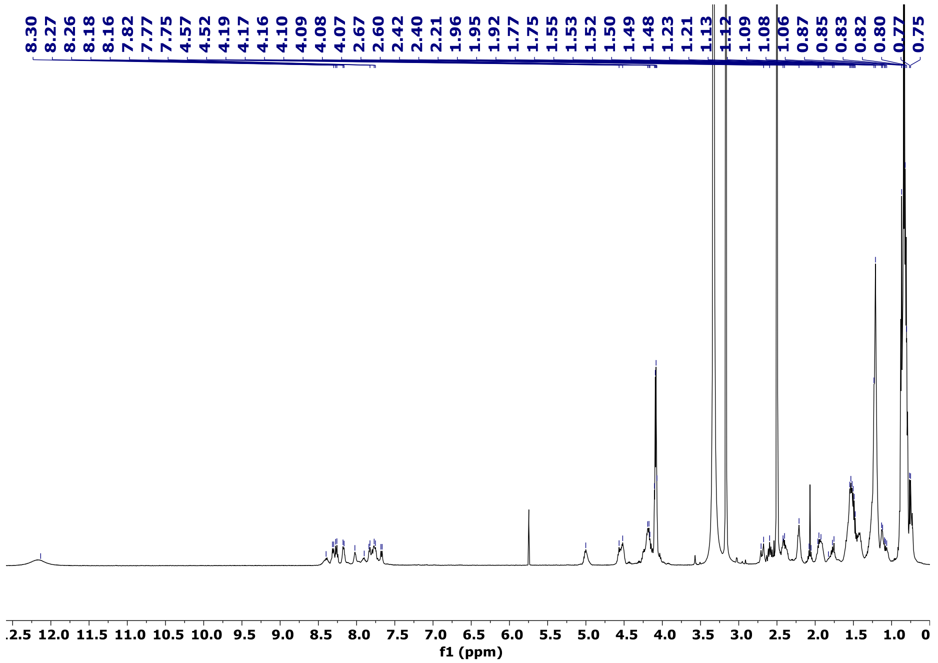

Figure S19: 500 MHz ^{13}C NMR spectrum of compound **1** in DMSO-d_6.

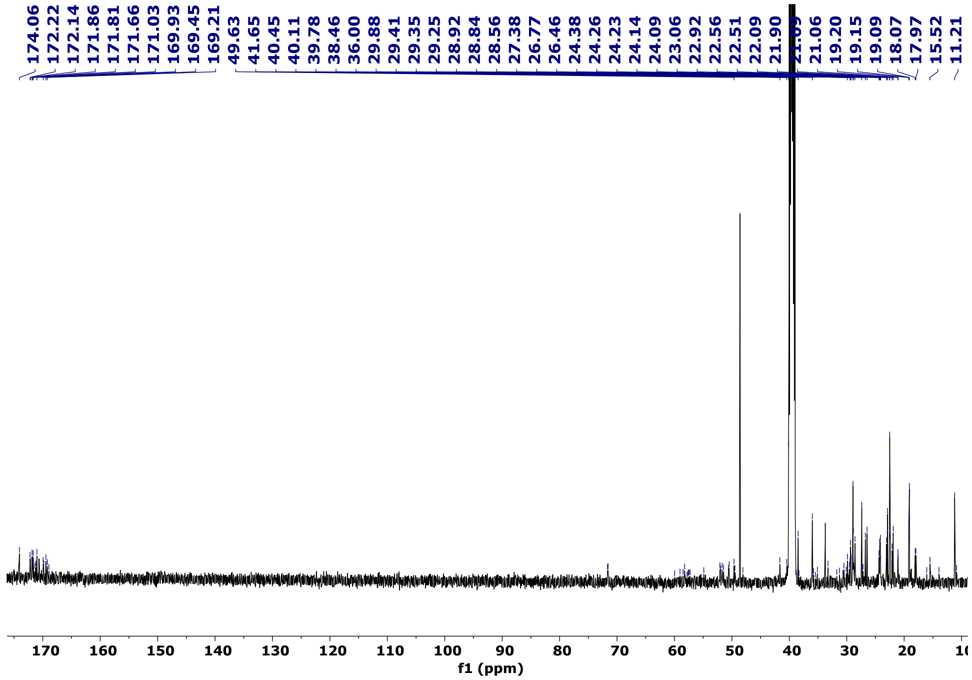

Figure S20: 500 MHz HSQC NMR spectrum of compound **1** in DMSO-d_6.

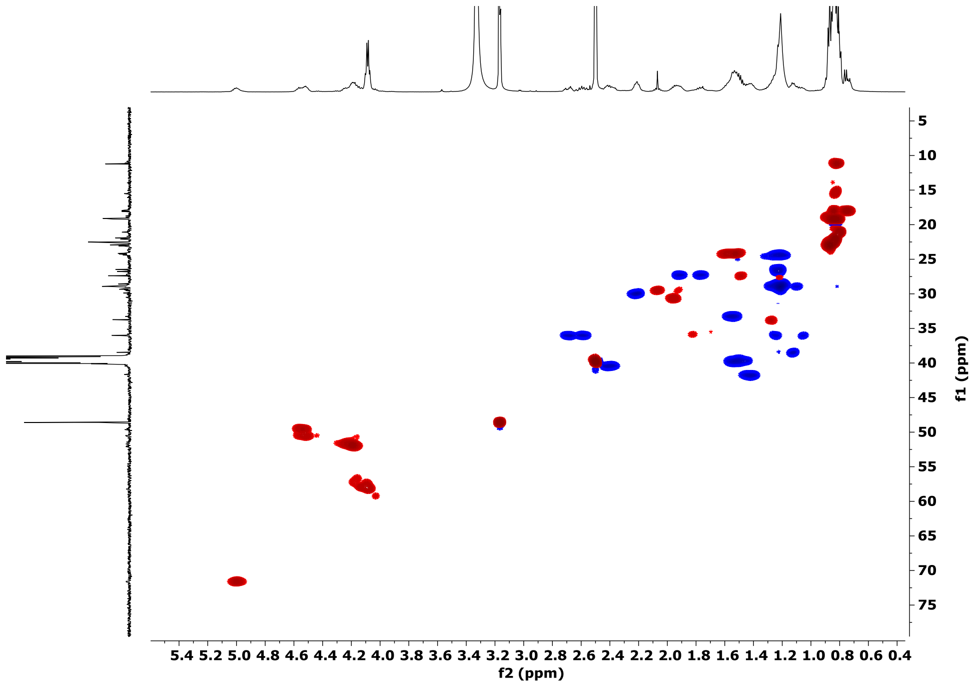

Figure S21: 500 MHz COSY NMR spectrum of compound **1** in DMSO-d_6.

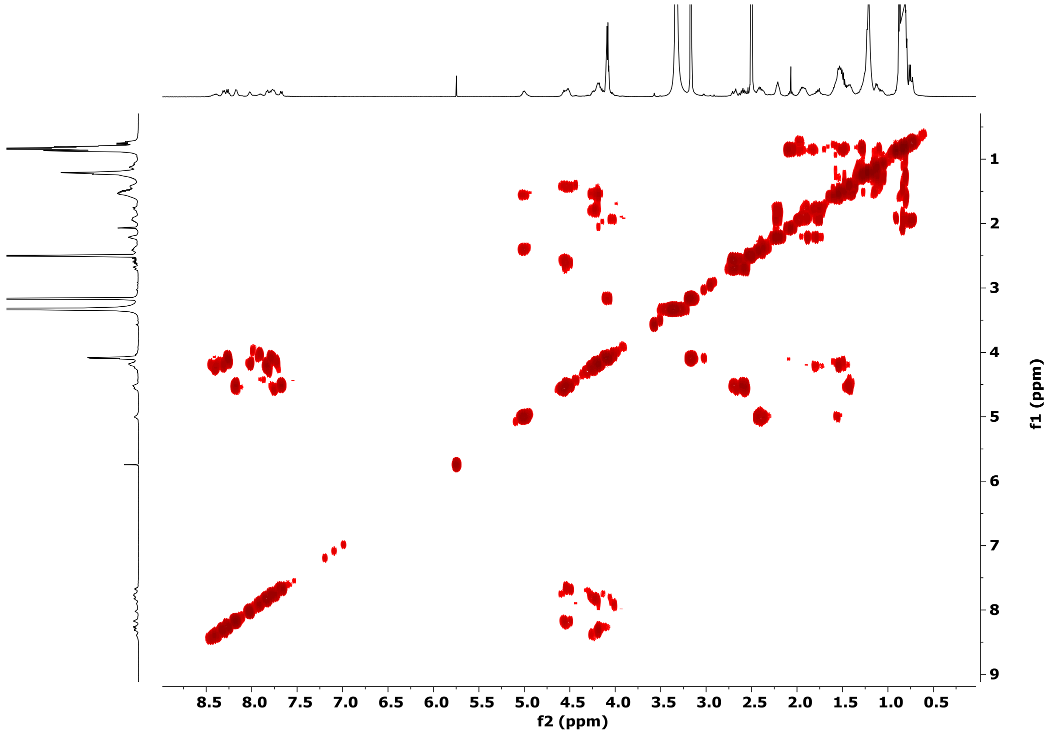

Figure S22: 500 MHz HMBC NMR spectrum of compound **1** in DMSO-d_6.

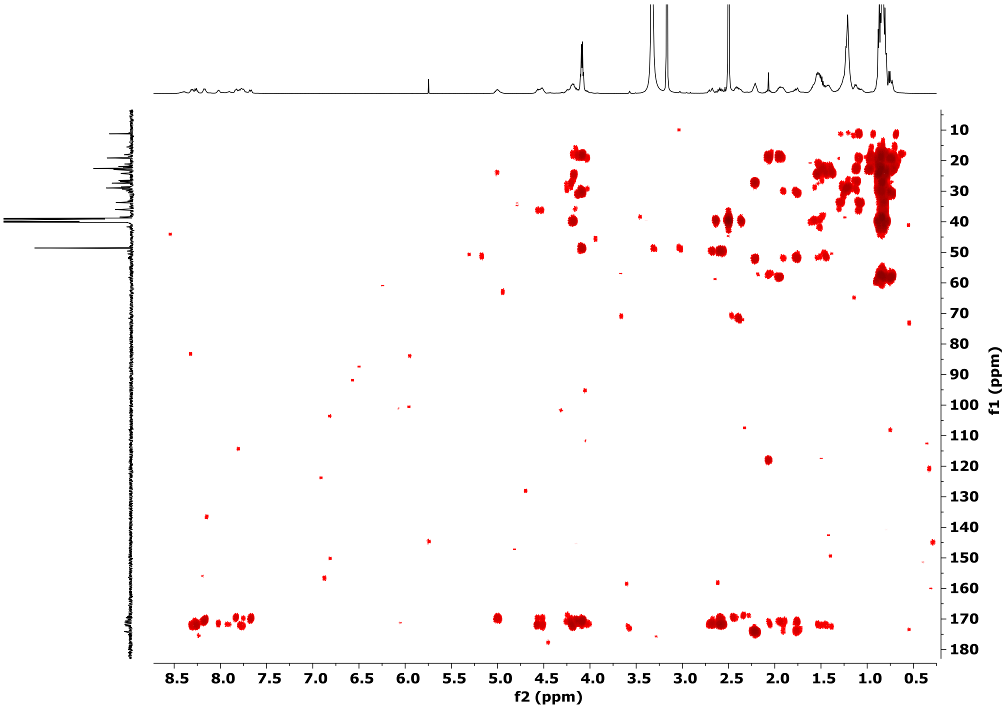

Figure S23: 500 MHz gHSQC-TOCSY NMR spectrum of compound **1** in DMSO-d_6.

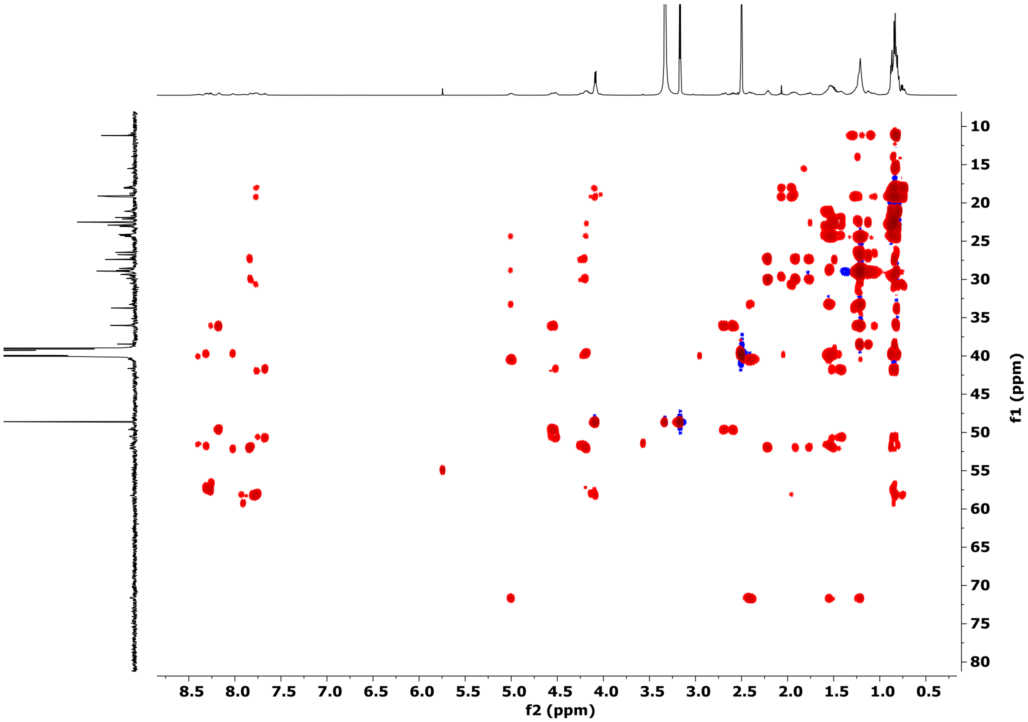

Figure S24: 500 MHz DEPT-135 NMR spectrum of compound **1** in DMSO-d_6.

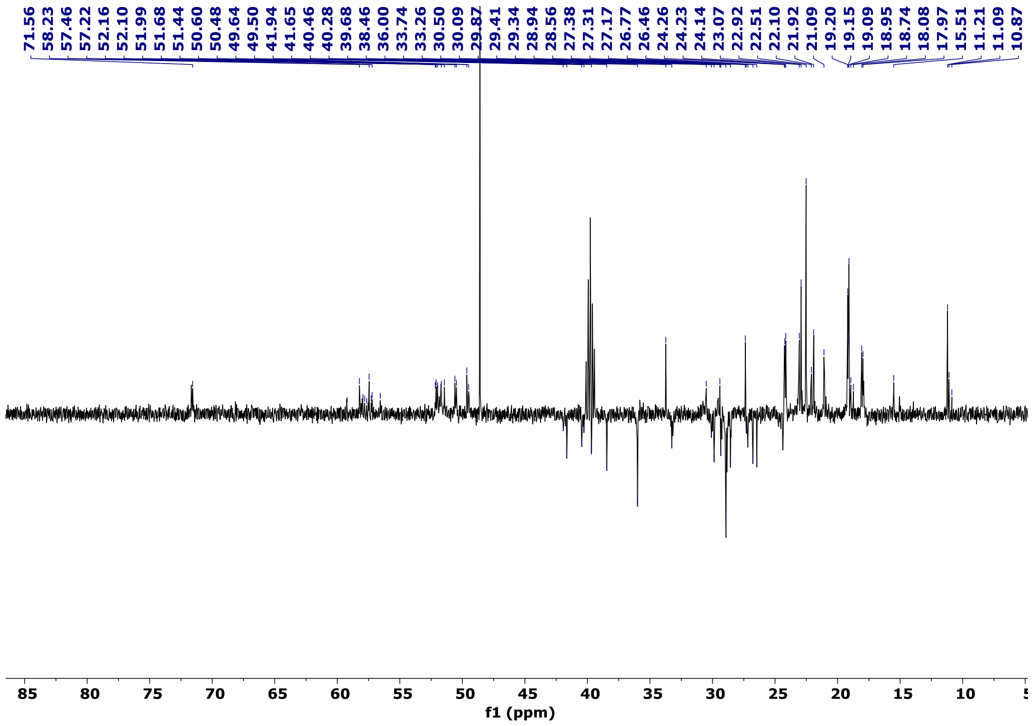

Figure S25. 500 MHz ROESY NMR spectrum of compound **1** in DMSO-d_6.

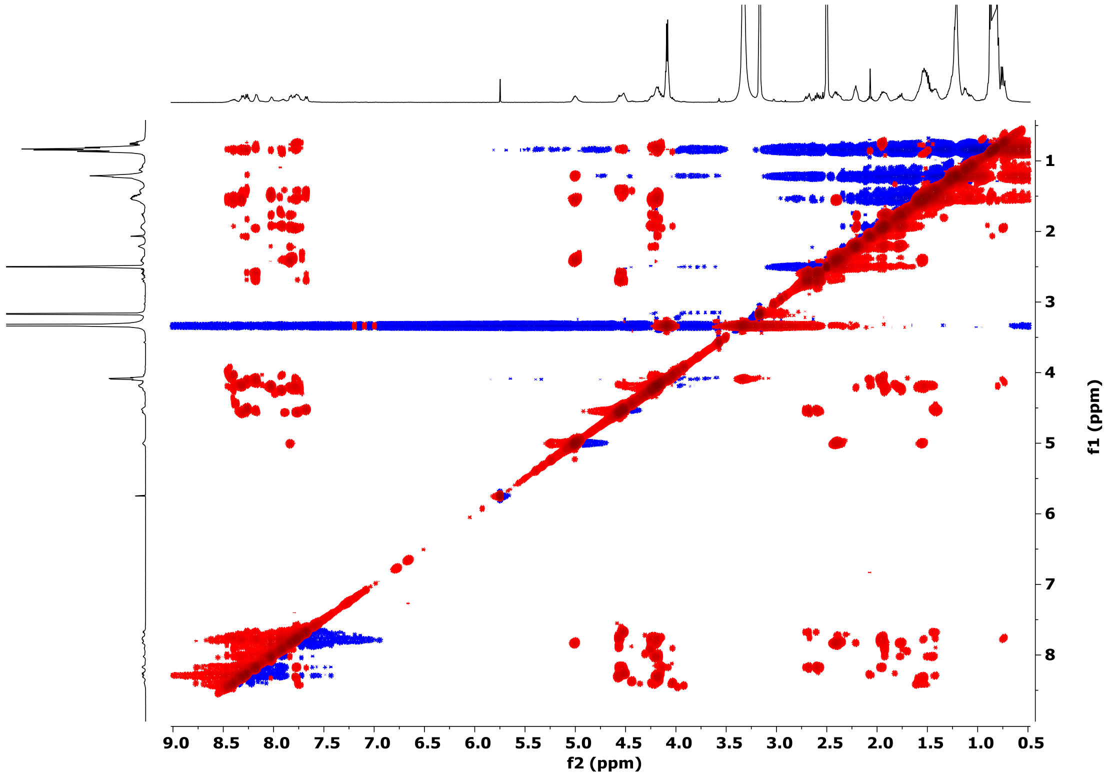

Figure S26: 500 MHz ^{1}H NMR spectrum of compound **1** in $CDCl_3$.

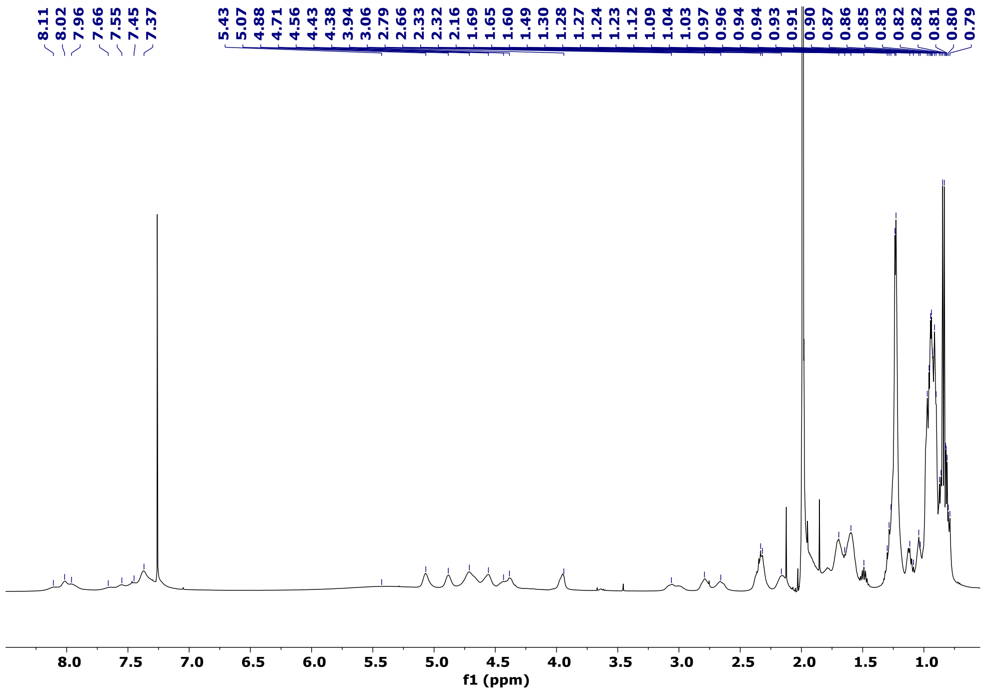

Figure S27: 500 MHz ^{13}C NMR spectrum of compound **1** in $CDCl_3$.

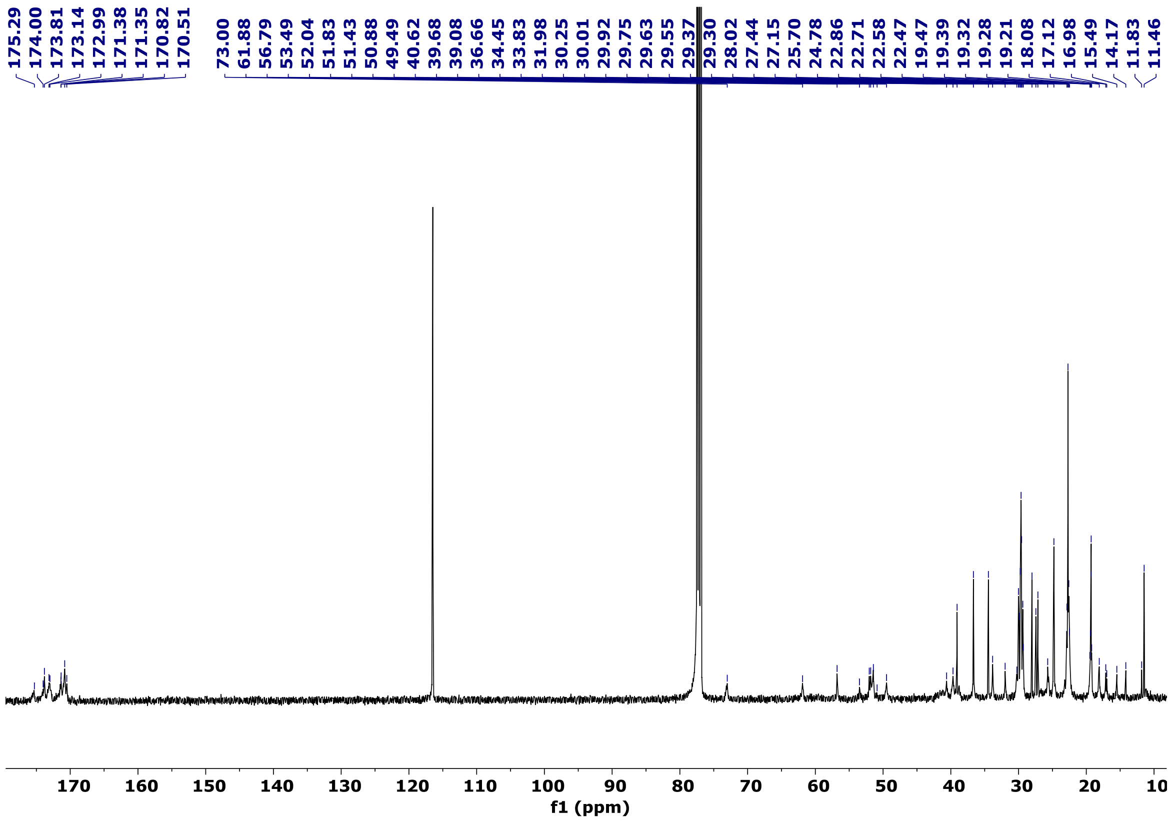

Figure S28: 500 MHz HSQC spectrum of compound **1** in $CDCl_3$.

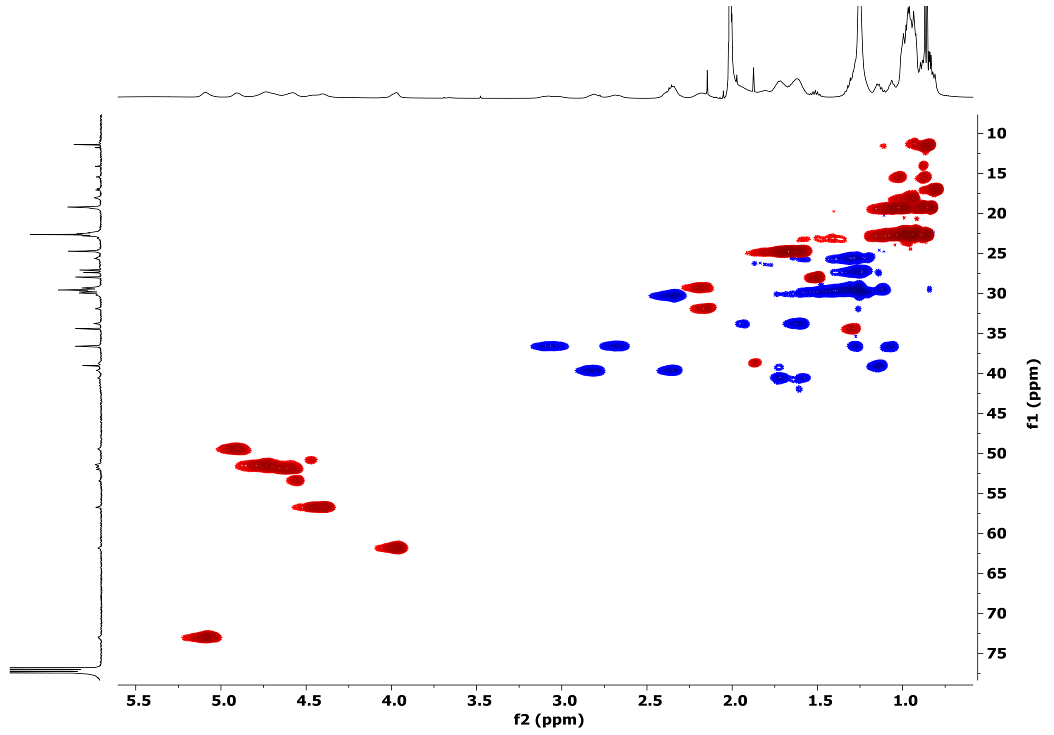

Figure S29: 500 MHz COSY spectrum of compound **1** in $CDCl_3$.

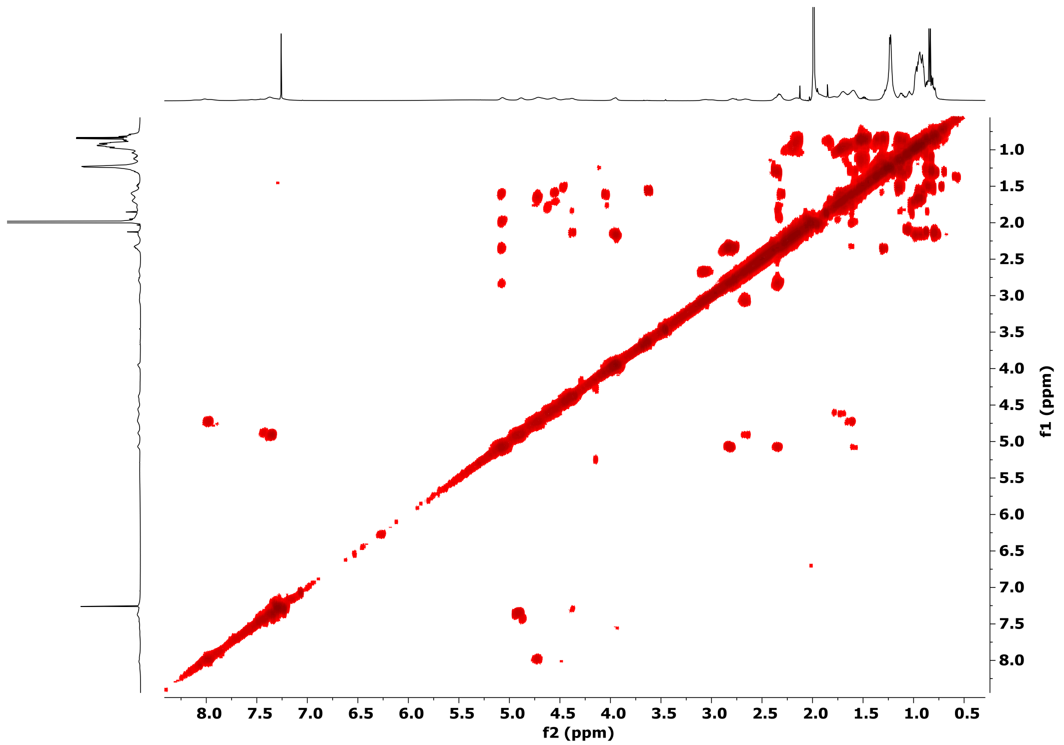

Figure S30: 500 MHz HMBC spectrum of compound **1** in $CDCl_3$.

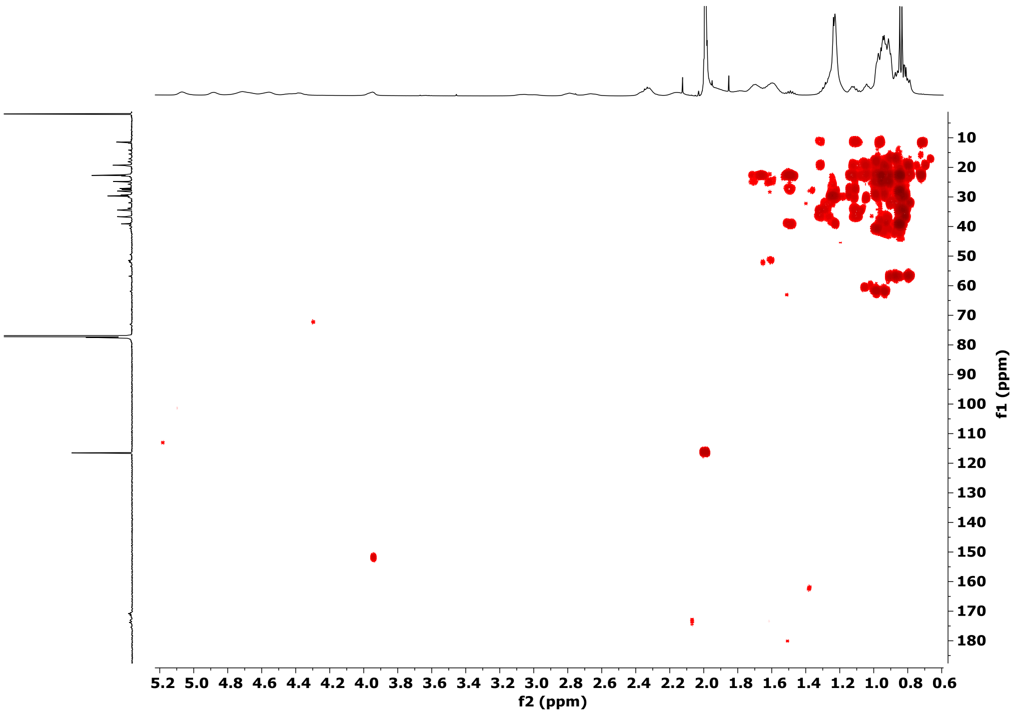

Figure S31: 500 MHz gHSQC-TOCSY spectrum of compound **1** in $CDCl_3$.

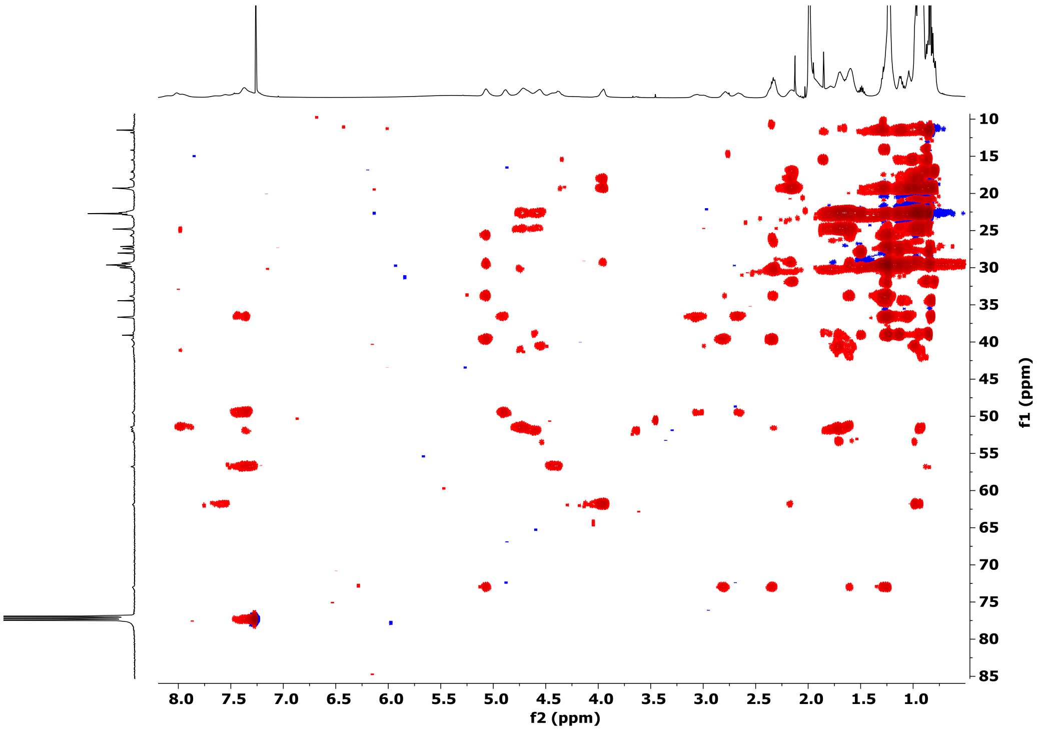

Figure S32: 500 MHz ROESY spectrum of compound **1** in $CDCl_3$.

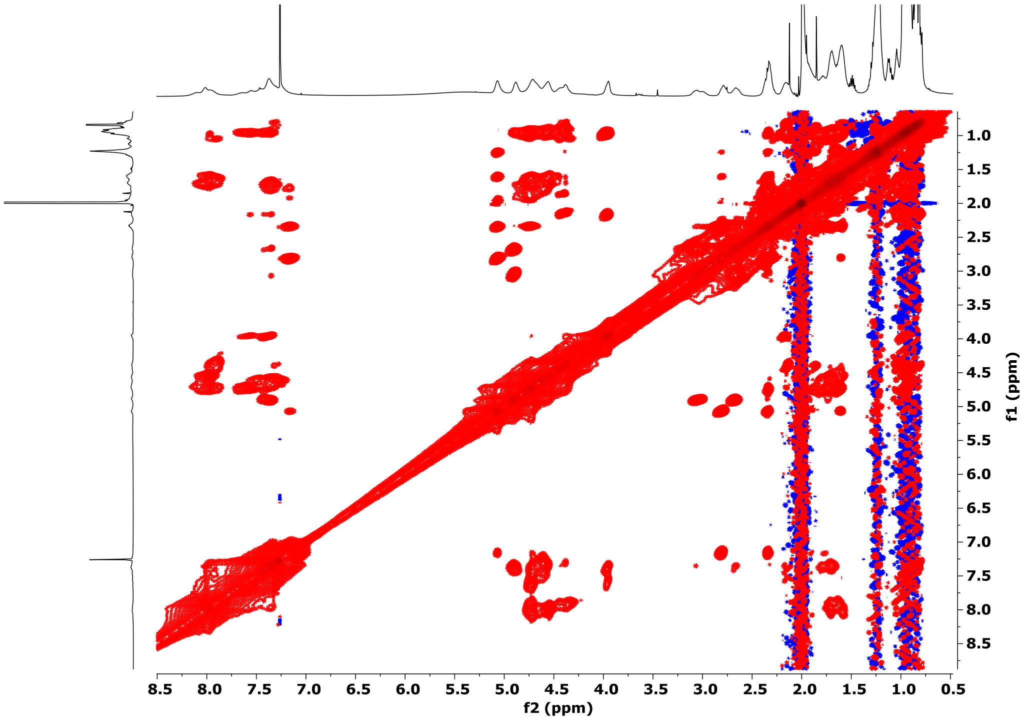

Figure S33: 500 MHz DEPT-135 spectrum of compound **1** in $CDCl_3$.

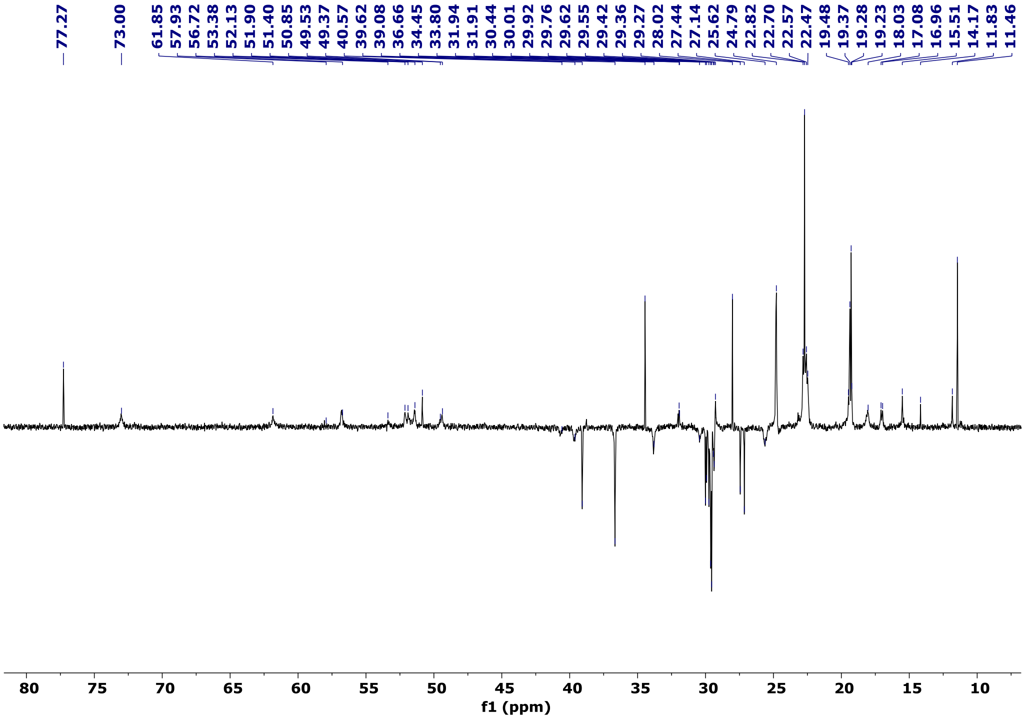

References

[1] Tetevi, G. M. *UGspace*, **2018**, http://ugspace.ug.edu.gh/handle/123456789/28431.

[2] Yoon, S. H.; Ha, S. M.; Kwon, S.; Lim, J.; Kim, Y.; Seo, H.; Chun, J. *Int. J. Syst. Evol. Microbiol.* **2017**, 67 1613–1617. doi:10.1099/ijsem.0.001755.

[3] Thompson, J. D.; Higgins, D. G.; Gibson, T. J. *Nucl. Acids Res.* **1994**, 22, 4673–4680.

[4] Kumar, S.; Stecher, G.; Tamura, K. *Mol. Biol. Evol.* **2016**, 33 1870–1874.

[5] Jukes, T. H.; Cantor, C. R. Evolution of Protein Molecules, in: Munro, H. N. (Eds.), Mammalian Protein Metabolism, Academic Press, New York, **1969**, pp. 21–132.

[6] Saitou, N.; Nei, M. *Mol. Biol. Evol*. **1987**, 4, 406–425.

[7] Felsenstein, J. *Ann. Rev. Ecol. Syst*. 1983, 14, 313–333.

[8] Felsenstein, J. *J. Mol. Evol*. **1981**, 17, 368–376.

[9] Felsenstein, J. *Evolution.* **1985**, 39, 783–791.

[10] Kupchan, M. S.; Britton, R. W.; Zeigler, M. F.; Sigel, C. W. *J. Org. Chem.* **1973**, 38, 178–179.

[11] Long, J.; Chen, Y.; Chen, W.; Wang, J.; Zhou, X.; Yang, B.; Liu, Y. Mar. Drugs 2021, 19, 701. https://doi.org/10.3390/md19120701

[12] Yabu, Y.; Minagawab, N.; Kitac, K. *Parasitol. Int.* **1998**, 48, 131–137.

[13] Osei, E.; Kwain, S.; Mawuli, G. T.; Anang, A. K.; Owusu, K. B. A.; Camas, M.; Camas, A. S.; Ohashi, M.; Alexandru-Crivac, C. N.; Deng, H.; Jaspars, M.; Kyeremeh, K. Mar. Drugs. 2019, 17, 9.

[14] Kwain, S.; Tetevi, G.M.; Mensah, T.; Camas, A. S.; Camas, M.; Dofuor, A. K.; Azerigyik, F. A.; Oluwabusola, E.; Deng, H.; Jaspars, M.; Kyeremeh, K. *Molbank*. **2019**, 3, M1080.
